# Complementary catalysis and analysis within solid state additively manufactured metal micro flow reactors

**DOI:** 10.1038/s41598-022-09044-9

**Published:** 2022-03-24

**Authors:** T. Monaghan, M. J. Harding, S. D. R. Christie, R. A. Harris, R. J. Friel

**Affiliations:** 1 School of Mechanical, Electrical and Manufacturing Engineering, Loughborough University, Loughborough, UK; 2 School of Chemical and Bioprocess Engineering, University College Dublin, Dublin, Ireland; 3 Department of Chemistry, Loughborough University, Loughborough, UK; 4 School of Mechanical Engineering, University of Leeds, Leeds, UK; 5 School of Information Technology, Halmstad University, Halmstad, Sweden

**Keywords:** Analytical chemistry, Catalysis, Chemical engineering, Electrical and electronic engineering, Mechanical engineering, Fluidics

## Abstract

Additive Manufacturing is transforming how researchers and industrialists look to design and manufacture chemical devices to meet their specific needs. In this work, we report the first example of a flow reactor formed via the solid-state metal sheet lamination technique, Ultrasonic Additive Manufacturing (UAM), with directly integrated catalytic sections and sensing elements. The UAM technology not only overcomes many of the current limitations associated with the additive manufacturing of chemical reactionware but it also significantly increases the functionality of such devices. A range of biologically important 1, 4-disubstituted 1, 2, 3-triazole compounds were successfully synthesised and optimised in-flow through a Cu mediated Huisgen 1, 3-dipolar cycloaddition using the UAM chemical device. By exploiting the unique properties of UAM and continuous flow processing, the device was able to catalyse the proceeding reactions whilst also providing real-time feedback for reaction monitoring and optimisation.

## Introduction

As a result of its notable advantages over its batch counterpart, flow chemistry is a significant and growing field in both academic and industrial settings due to its ability to enhance the selectivity and efficiency of chemical synthesis. This extends from simple organic molecule formation^1^, to pharmaceutical compounds^2,3^ and natural products^4–6^. Upwards of 50% of the fine chemical and pharmaceutical sectors' reactions could benefit from the adoption of continuous flow processing^7^.

In recent years, a growing trend has emerged in which groups have looked to replace traditional glassware or flow chemistry equipment in favour of customisable, Additively Manufactured (AM) chemical 'reactionware'^8^. The iterative design, rapid production, and 3-Dimensional (3D) capabilities of these technologies are highly beneficial to those looking to tailor their device to a particular set of reactions, equipment, or conditions. To date, this work has almost exclusively focused on the use of polymer-based 3D printing techniques such as Stereolithography (SL)^9–11^, Fused Deposition Modelling (FDM)^8,12–14^ and Inkjet Printing^7,15,16^. Such devices lack robustness and the ability to perform a wide range of chemical reactions/analysis^17–20^, which has been a major limiting factor in the greater implementation of AM in this area^17–20^.

As a result of the growing use of flow chemistry and the advantageous properties associated with AM, it is pertinent to explore more advanced technologies which allow the user to manufacture flow-reaction ware with increased chemical and analytical functionality. These techniques should enable a user to select from a range of highly robust or functional materials capable of dealing with a wide range of reaction conditions while also facilitating various analytical output forms from the device to permit reaction monitoring and control.

One AM process with the potential to develop bespoke chemical reactionware is Ultrasonic Additive Manufacturing (UAM). This solid-state sheet lamination technology applies ultrasonic oscillations to thin metallic foils in order to join them together, layer-by-layer, with minimal bulk heating and high degrees of plastic flow^21–23^. Unlike most other AM techniques, UAM can integrate directly with subtractive manufacturing, termed a hybrid manufacturing process, in which *in-situ* periodic computer numerical controlled (CNC) milling or laser processing defines the net shape of the bonded material layers^24,25^. This means the user is not restricted by issues associated with removing residual unprocessed build materials from small fluidic pathways, as is often the case with powder and liquid AM systems^26–28^. This design freedom also extends to the material choices available—UAM can bond thermally similar and dissimilar material combinations in a single process step. Material combination choices beyond those of melt processes mean that one can better meet the mechanical and chemical needs of a particular application. In addition to solid-state bonding, an additional phenomenon encountered during ultrasonic bonding is a high degree of plastic material flow at relatively low temperature^29–33^. This unique feature of UAM can facilitate the embedment of mechanically/thermally sensitive elements between metal layers without damage. UAM embedded sensors could facilitate the delivery of real-time information from the device to the user via integrated analytics.

Past work by the authors^32^ showed the ability of the UAM process to create metallic 3D microfluidic structures with integrated sensing; this was a monitoring only device. This paper presents the first example of a microfluidic chemical reactor manufactured via UAM; this is an active device that not only monitors but also induces chemical synthesis via structurally integrated catalyst material. This device combines several of the advantages associated with UAM technology in 3D chemical device manufacture, such as; the ability to translate a fully 3D design directly from a computer-aided design (CAD) model to a product; multi-material manufacture to incorporate high thermal conductivity and catalytic materials; and embedded thermal sensors directly between reagent streams for precise reaction temperature monitoring and control. To demonstrate the reactors functionality, a library of pharmaceutically important 1,4-disubstituted 1, 2, 3-triazole compounds were synthesised via a copper-catalysed Huisgen 1,3-dipolar cycloaddition. The work highlights how the utilisation of material science and computer-aided design can open new opportunities and possibilities in chemistry through multi-disciplinary research.

## Materials and methods

### General

All solvents and reagents were purchased from Sigma-Aldrich, Alfa Aesar, TCI, or Fischer Scientific, and were used without prior purification. ^1^H and ^13^C NMR spectra, recorded at 400 MHz and 100 MHz respectively, were obtained using a JEOL ECS-400 400 MHz spectrometer or Bruker Avance II 400 MHz spectrometer and CDCl_3_ or (CD_3_)_2_SO as a solvent. All reactions were performed using a Uniqsis FlowSyn flow chemistry platform.

### Flow reactor fabrication

**Figure 1:**
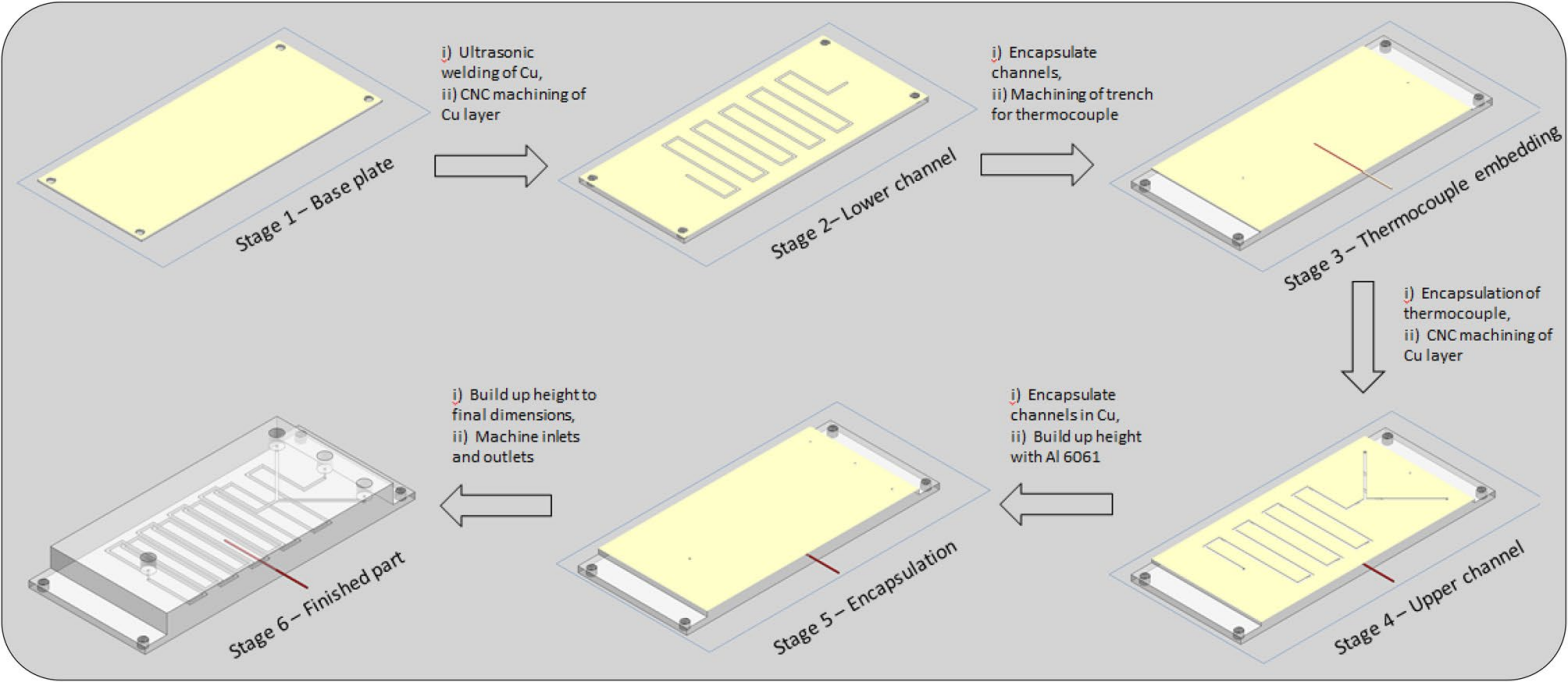
Stages of reactor manufacture (1) Al 6061 base plate (2) Machining of bottom channel set into copper foil (3) Embedding of thermocouple between layers (4) Top channel Sect. (5) Inlets and outlet (6) Overall reactor.

UAM was used to manufacture all devices in this study. The technology was invented in 1999 and the technology details, operational parameters and developments since its invention can be studied with the following published sources^34–37^**.** The device (Fig. 1) was achieved using an ultra-high power, 9kW SonicLayer 4000® UAM system (Fabrisonic, OH, USA). The materials chosen for the manufacture of the flow device were Cu-110 and Al 6061. Cu-110 has a high Cu content (min. 99.9% Cu), making it a good candidate for Cu catalysed reactions and was thus used as the 'active' layers within the micro-reactor. Al 6061 O was used as the 'bulk' material and also as the embedding layers for the analytics; the annealed state of the alloy aided component embedment and bonding with the Cu-110 layers. Al 6061 O is a material that has been shown to be highly compatible with the UAM process^38–41^ and was tested and found to be chemically stable with the reagents used in this work. The bonding of the Al 6061 O to Cu-110 was also known to be a compatible material combination for UAM and thus was a suitable material for this study^38,42^ The welding parameters used for the ultrasonic consolidation of each material combination encountered within the layering of the devices are located below in Table 1.

**Table 1:** Ultrasonic additive manufacturing (UAM) processing parameters used for bonding the various Al and Cu material combinations. (Due to the UAM process dynamics, parameters must be adjusted as the height of the part increases to account for an increased height to width ratio^40,43^).

Material Combination	Force [N]	Speed [mm/s]	Amplitude [µm]	Temp (°C)
Al-Al	4000	85	34	82
Cu-Al	4000	64	36	82
Cu-Cu	4000	64	39	66
Al-Cu	5000	85	34	77
Al-Al	4000	85	33	77

The design philosophy for the fluidic pathways was to use a convoluted pathway to increase the distance within the chip that the fluid travelled whilst keeping the chip to a manageable size. This distance increase is desirable to increase catalyst/reagent interaction time and provide a superior product yield. The chip used 90° bends at the ends of the straight pathways to induce turbulent mixing^44^ within the device and increase the fluid to surface (catalyst) contact time. To further increase the mixing that could be achieved, the reactor was designed to feature two reagent inlets combining at a Y-junction before entering the serpentine mixing section. A third inlet intersecting the stream halfway through its residence was included in the design for future multi-step reaction synthesis.

All channels had a square profile (no draft angle) which is a result of the periodic CNC milling that was used to create the channel geometries. The channel dimensions were chosen to ensure high (for a micro-reactor) volume output whilst being small enough to encourage surface interaction (catalyst) for the majority of the contained fluid. A suitable size was based on past research experience of the authors with metallic fluidic devices for reactions. The final channels had an internal size of 750 µm x 750 µm yielding a total reactor volume of 1 ml. Integrated connectors (1/4"—28 UNF thread) were included in the design to allow for simple interfacing of the devices with commercial flow chemistry apparatus. The channel size is limited to the thickness of the foil material, its mechanical properties and the ultrasonic bonding parameters used. At a certain width for a given material the material will ‘sag’ into the channel that has been created. There is currently no specific model for this calculation and thus maximum channel widths for the given materials and design are determined experimentally; in this instance the 750 μm width did not result in sagging.

The shape of the channel (square) was determined by the use of a square cutting mill for the channels. The shape and size of the channels can be altered using a different cutting tool with the CNC machine to attain different flow rates and characteristics. An example that used a 125 μm tool to create curved shaped channels can be found in the work by Monaghan^45^. The capping layer of foil material over the channel will have a straight (square) finish as the foil layers are deposited in a planar fashion. In this work, to maintain symmetry of the channels a square profile was used.

During pre-programmed pauses in the manufacture, a thermocouple temperature probe (Type K) was directly embedded between the upper and lower sets of channels within the device (Fig. 1—Stage 3). These thermocouples can monitor temperature changes from − 200 to 1350 °C.

The metal deposition process is performed by the UAM sonotrode using 25.4 mm wide metal foils that are 150 μm thick. These foil layers are bonded in a series of adjacent strips to cover the entire build area; the deposited material is larger in size than the final product as a subtractive process creates the final net shape. CNC machining is used to machine the external and internal contours of the device thus achieving a surface finish of the device and channels that is equal to the tool and CNC process parameters chosen (approx. 1.6 μm Ra in this instance). Continuous, sequential, ultrasonic material deposition and machining cycles are used during the entire device manufacturing process to ensure dimensional accuracy is maintained and the finished component will meet CNC finish milling levels of accuracy. The channel width used for this device is sufficiently small to ensure the foil material does not ‘sag’ into the fluidic channel and thus the channel maintains a square cross-section. The gap possible with the foil material and UAM process parameters was experimentally determined by the manufacturing partner (Fabrisonic LLC, USA).

Studies have shown that little elemental diffusion occurs at the UAM bond interfaces^46,47^ without additional heat treatment and thus for the devices in this work the Cu-110 layers remain distinct and abruptly change from the Al 6061 layers.

### Preliminary reactor testing

A pre-calibrated 250 psi (1724 kPa) Back Pressure Regulator (BPR) was fitted to the reactor's outlet and water pumped through the reactor at 0.1 to 1 mL min^-1^. The reactor pressure was monitored using the FlowSyn inbuilt system pressure sensor to verify the system could maintain a constant steady pressure. Possible temperature gradients across the flow reactor were tested by establishing any differences between the thermocouple embedded within the reactor and the thermocouple embedded within the FlowSyn chip heating plate. This was achieved by varying the programmable hotplate temperature between 100 and 150 °C in 25 °C increments and noting any differences between programmed and recorded temperature. This was achieved using a tc-08 data logger (PicoTech, Cambridge, UK) and accompanying PicoLog software.

### Model triazole reaction optimisation

**Scheme 1:**
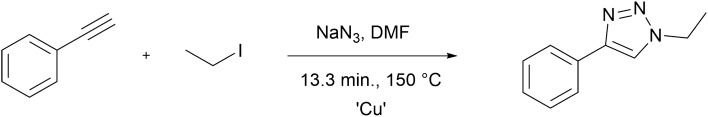
Cycloaddition of Phenylacetylene and Iodoethane.

An optimisation of reaction conditions was performed with respect to the cycloaddition of phenylacetylene and iodoethane (Scheme 1—Cycloaddition of Phenylacetylene and IodoethaneScheme 1—Cycloaddition of Phenylacetylene and Iodoethane). This optimisation was carried out through a full factorial Design of Experiments (DOE) approach, using both temperature and residence as variable parameters whilst keeping the alkyne:azide ratio fixed at 1:2.

Separate solutions of sodium azide (0.25 M, 4:1 DMF:H_2_O), iodoethane (0.25 M, DMF) and phenyl acetylene (0.125 M, DMF) were prepared. 1.5 mL aliquots of each solution were mixed and pumped through the reactor at the desired flow rate and temperature. The model response was taken as the peak area ratio of the triazole product to the phenylacetylene starting material, as determined by high-performance liquid chromatography (HPLC). All reactions were sampled just after the reaction mixture exited the reactor, for consistency in analysis. The parameter ranges chosen for the optimisation are shown in Table 2.

**Table 2:** Design of Experiments (DOE) parameters used in reaction optimisation.

Variable	Low	Centre Point	High
Temperature [^o^C]	100	125	150
Residence Time [min.]	5	10	15

### HPLC analysis of DOE samples

All samples were analysed using a Chromaster HPLC system (VWR, PA, USA) consisting of a quaternary pump, column oven, variable wavelength UV detector and an autosampler. The column was an Equivalence 5 C18 (VWR, PA, USA) with 4.6 × 100 mm dimensions, and a 5 µm particle size held at 40 °C. The solvent was isocratic 50:50 methanol:water with a flow rate of 1.5 mL.min^-1^. The injection volume was 5 µL, and the detector wavelength was 254 nm. The % peak area for DOE samples was calculated from the peak areas of the residual alkyne and the triazole product only. Injections of the starting materials allowed the identification of relevant peaks.

Coupling the reactors analytical output to MODDE DOE software (Umetrics, Malmö, Sweden), it was possible to thoroughly analyse resulting trends and establish the optimal reaction conditions for this cycloaddition. Running an inbuilt optimisation program with all significant model terms selected enabled the generation of a set of reaction conditions intended to maximise product peak area whilst subsequently reducing the acetylene starting material peak area.

### Hydrogen peroxide surface oxidation treatment

Oxidation of surface copper within the catalytic reaction chamber was achieved using a solution of Hydrogen peroxide (36%) flowed through the reaction chamber (Flow Rate = 0.4 mL min^-1^, Residence Time = 2.5 min) prior to each triazole compound library synthesis.

### Triazole library synthesis procedure

**Figure 2:**
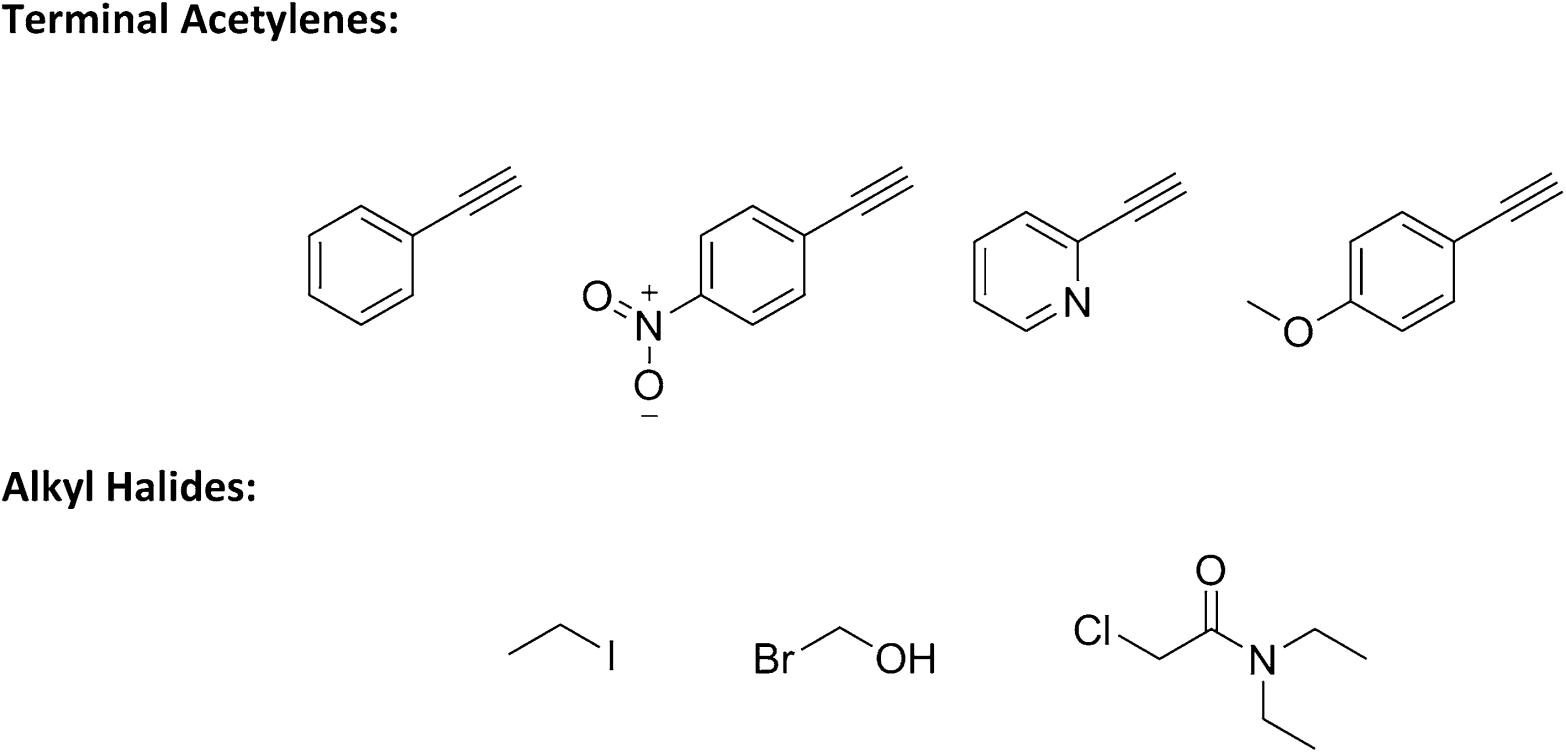
Acetylene and Alkyl Halides derivatives used in library synthesis.

Once an optimal set of conditions had been established, they were applied to a range of acetylene and alkyl halides derivatives to allow a small library synthesis to be compiled, thus establishing the ability to apply these conditions to a broader range of potential reagents (Fig. 2).

Separate solutions of sodium azide (0.25 M, 4:1 DMF:H_2_O), alkyl halide (0.25 M, DMF) and alkyne (0.125 M, DMF) were prepared. 3 mL aliquots of each solution were mixed and pumped through the reactor at 75 µL.min-1 and 150 °C. The total volume was collected into a vial and diluted with 10 mL ethyl acetate. The sample solution was washed with 3 × 10 mL of water. The aqueous layers were combined and extracted with 10 mL of ethyl acetate; the organic layers were then combined, washed with 3 × 10 mL of brine, dried with MgSO_4_ and filtered before the solvent was removed in vaccuo. Samples were purified by column chromatography on silica gel using ethyl acetate prior to analysis via a combination of HPLC, ^1^H NMR, ^13^C NMR and high-resolution mass spectroscopy (HR-MS).

### High-Resolution mass spectra

All spectra were obtained using a Thermofischer exactive Orbitrap resolution mass spectrometer, with ESI as the ionisation source. All samples were prepared using Acetonitrile as the solvent.

### Thin layer chromatography

TLC analysis was performed on aluminium backed silica plates. Plates were visualised by ultraviolet light (254 nm), or with vanillin stain and heating.

### Liquid chromatography/Mass spectroscopy analysis

All samples were analysed using a VWR Chromaster (VWR International Ltd., Leighton Buzzard, UK) system equipped with an autosampler, column oven binary pump and single wavelength detector. The column used was an ACE Equivalence 5 C18 (150 × 4.6 mm, Advanced Chromatography Technologies Ltd., Aberdeen, Scotland).

Injections (5µL) were made directly from diluted crude reaction mixtures (1 in 10 dilution) and analysed with water: methanol (50:50 or 70:30), except for some samples which were analysed with a 70:30 solvent system (denoted by an asterisk), at a flow rate of 1.5 mL/min. The column was held at 40 °C. The detector wavelength was 254 nm.

The % peak area for samples was calculated from the peak areas of the residual alkyne, and the triazole product only, injections of the starting materials allowed the identification of relevant peaks.

### Inductively coupled plasma-optical emission spectroscopy

All samples were analysed using a Thermo iCAP 6000 ICP-OES. All calibration standards were prepared using a 1000 ppm Cu standard solution in 2% nitric acid (SPEX Certi Prep). All standards were prepared in 5% DMF and 2% HNO_3_ solution, and all samples were diluted by a factor of 20 in the sample DMF—HNO_3_ solution.

## Theory

### UAM bonding theory

UAM utilises ultrasonic metal welding as the bonding technique for the metal foil materials used to construct the final component. Ultrasonic metal welding utilises an oscillating metal tool (known as a horn or sonotrode) to apply pressure to the foil layer/previously consolidated layers to be bonded, whilst simultaneously oscillating the material. To work continuously, the sonotrode is cylindrical and rolls over the material's surface, bonding the whole area. When the pressure and oscillation is applied, the oxides on the materials surface break-up. The continued pressure and oscillation then cause the asperities of the material to collapse^36^. The intimate contact with the locally induced heat and pressure then leads to a solid-state bond occurring at the material interface; it may also be aided through changes in surface energy aiding in adhesion^48^. The nature of the bonding mechanism overcomes many of the issues associated with variable melt temperatures and high temperature after effects noted in other additive manufacturing techniques. This allows the direct bonding (i.e., without surface modification, fillers or adhesives) of multiple dissimilar material layers into a single consolidated structure.

The secondary advantageous factor in UAM is the high degree of plastic flow observed in the metal material, even at low temperatures, i.e., considerably lower than the metal's material melting point. The combination of ultrasonic oscillation and pressure induce a high level of, localised, grain boundary mobility and recrystallisation without the traditionally associated large increase in bulk material temperature. This phenomenon can be exploited to embed active and passive elements between the metal foil layers during the construction of the final component, layer by layer. Elements such as optical fibres^49^, strengthening reinforcement^46^, electronics^50^ and thermocouples (this work) have all been successfully embedded into UAM structures to create active and passive composite material components.

Both the dissimilar material bonding and embedding possibilities of UAM have been utilised in this work to create the final catalytic temperature monitoring micro-reactor.

### Copper catalysis

Cu catalysis exhibits several advantages over Palladium (Pd) and other commonly used metal catalysts: (i) Economically, Cu is cheaper than many of the other metals used in catalysis and is, therefore, an attractive option within the chemical processing industry (ii) the scope of Cu-catalysed cross-coupling reactions is increasing and appears to be somewhat complementary to that of Pd-based methodologies^51–53^ (iii) Cu-catalysed reactions work well in the absence of additional ligands, and if required, these ligands are often structurally simple and inexpensive whereas ligands for Pd chemistry are often complex, expensive and air-sensitive (iv) Cu, in particular, has been known for its ability to bind to alkynes in synthesis, e.g. bimetallic-catalysed Sonogashira coupling and the cycloaddition reaction with azides (click chemistry) (v) Cu is also able to promote arylation reactions of several nucleophilic species in Ullmann type reactions^54^.

Heterogenised examples of all of these reactions have recently been demonstrated in the presence of Cu(0). This is in no small part due to the pharmaceutical industry and the increasing attention on recycling and reuse of metal-based catalysts^55,56^.

### Huisgen 1, 3 dipolar cycloaddition

Pioneered by Huisgen in the 1960s^57^, the 1,3-dipolar cycloaddition reaction between acetylenes and azides to yield 1,2,3-triazoles is regarded as a concerted exemplar reaction. The resulting 1,2,3 triazole moiety is of particular interest as a pharmacophore in the field of drug discovery as a result of their biological applications and use in a variety of therapeutic agents^58^.

This reaction was brought back into focus by Sharpless and others when they developed the concept of "Click Chemistry"^59^. The 'Click Chemistry' term is used to describe a set of powerful, reliable and selective reactions for the rapid synthesis of new compounds and combinatorial libraries via heteroatom links (C-X-C)^60^ The synthetic appeal of these reactions' stems from their associated high yields, simple reaction conditions, tolerance of oxygen and water, and simple product isolation^61^.

**Figure 3:**
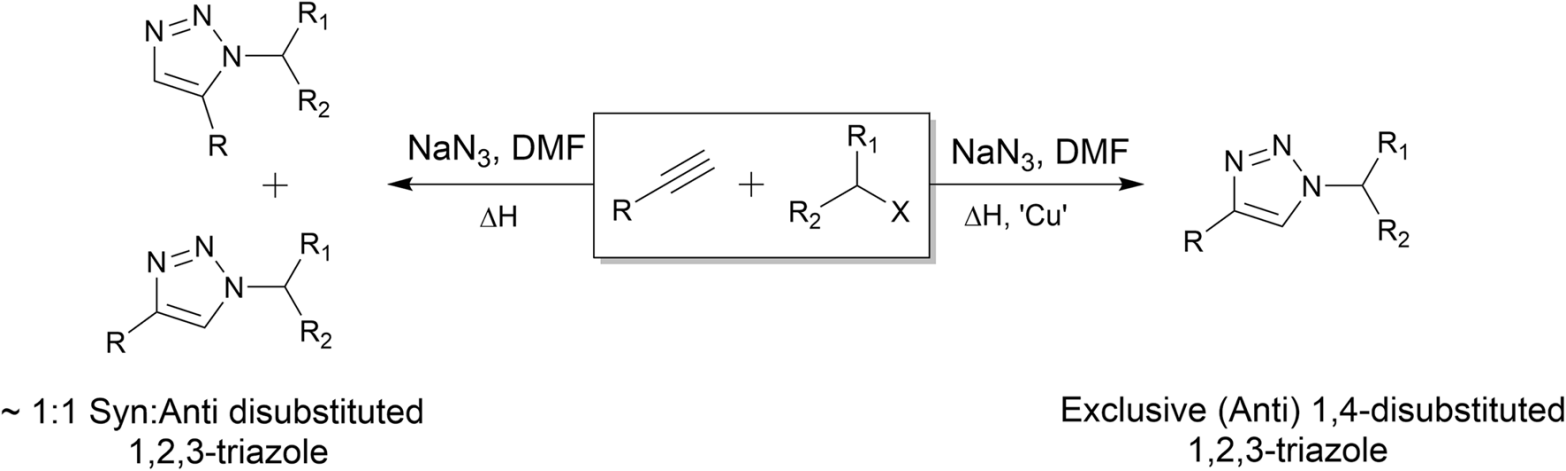
Isomeric outcome of both traditional and copper catalysed Huisgen cycloadditions. The Cu(I) catalysed Huisgen cycloaddition results in exclusive formation of the 1,4-disubstituted 1,2,3-triazole whilst the thermally induced Huisgen cycloaddition typically yields a 1:1 mixture of 1,4- and 1,5-trizole stereoisomers.

The classical Huisgen 1,3-dipolar cycloaddition does not fall into the 'Click Chemistry' class. However, Medal and Sharpless demonstrated that this azide–alkyne coupling event experiences dramatic rate acceleration of 10^7^ to 10^8^ in the presence of Cu(I) when contrasted with the uncatalysed 1,3-dipolar cycloaddition^62,63^. This modified reaction mechanism requires no protecting groups or harsh reaction conditions and proceeds with almost complete conversion and selectivity for the 1,4-disubstituted 1,2,3-triazole (anti-1,2,3- triazole) in mild conditions and short timescales (Fig. 3).

The majority of protocols involve either the reduction of stable sources of Cu(II) such as CuSO_4_ with sodium salts or the coproportionation of Cu(II)/Cu(0) species. Compared with other metal-catalysed reactions, the use of Cu(I) presents the major advantages of being inexpensive and easy to handle^64^.

Kinetic and isotopic labelling studies performed by Worrell et al.^65^ have shown that, in the case of terminal alkynes, two equivalents of copper participate in activating each molecule's reactivity toward azide. The proposed mechanism proceeds via a six-membered copper metallacycle, formed through coordination of an azide to a σ-bound copper acetylide bearing a π-bound copper which acts as a stabilising donor ligand. A triazolyl-copper derivative is formed through ring contraction, followed by protonolysis to deliver the triazole product and close the catalytic cycle.

## Results and discussion

### UAM Flow Reactor

**Figure 4:**
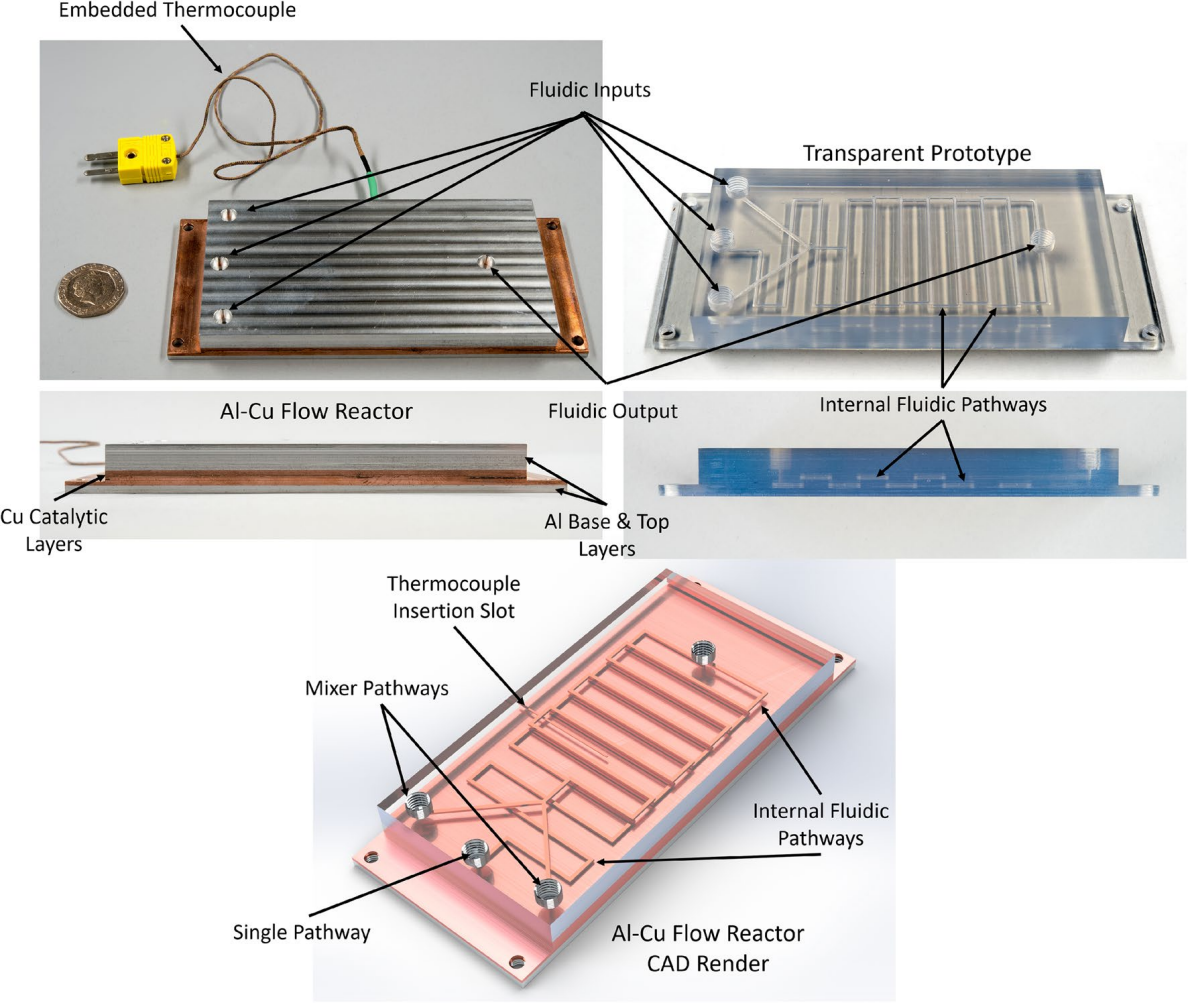
Al-Cu Flow Reactor manufactured via Ultrasonic Additive Manufacturing (UAM), featuring complex internal channel structures, embedded thermocouple and the catalytic reaction chamber. For visualisation of the internal fluidic pathway, a transparent prototype manufactured using Stereolithography is also shown.

While the merits of flow chemistry devices are well documented, there is a continuous desire to integrate analytical tools within these systems for online, in-situ, process monitoring^66^^,^^67^. UAM was demonstrated as a suitable method for the design and production of highly complex 3D flow reactors from catalytically active, thermally conductive materials featuring directly embedded sensing elements (Fig. 4).

To ensure the fabricated reactors were suitable for future organic reactions, solvents would need to be safely heated above their boiling point; they were pressure and temperature tested. Pressure testing demonstrated that even at elevated system pressures (1.7 MPa), the system maintained a steady and constant pressure. The static pressure testing was performed at room temperature using H_2_O as the fluid.

Attaching the embedded (Fig. 1) thermocouple to the temperature data logger demonstrated that the thermocouple's temperature was 6 °C (± 1 °C) less than the temperature-programmed on the FlowSyn System. Typically, a 10 °C increase in temperatures leads to a doubling of reaction rate, and therefore temperature discrepancies of only a few degrees can significantly alter reaction rates. This difference was attributed to temperature loss across the body of the reactor due to the high thermal diffusivity of the materials used in manufacture. This thermal drift was consistent and so could be accounted for in the device setup, to ensure an accurate temperature was being attained and measured during the reaction. Therefore, this in-line monitoring tool facilitated tight control over the reaction temperatures and facilitated more accurate process optimisation and development of optimal conditions. These sensors could also be useful in identifying reaction exotherms and preventing reaction runaway in larger-scale systems.

The reactor presented in this work is the first example of UAM technology being applied to the manufacture of chemical reactors and addressed several of the major limitations currently associated with the AM/3D printing of these devices, such as; (i) overcoming issues associated with the processing of Cu or Al alloys as reported in SLM^68^ (ii) enhanced internal channel resolution when compared with powder bed fusion (PBF) techniques, e.g. selective laser melting (SLM)^25,69^ as issues associated with poor flowing nature of the feedstock material and rough surface textures are removed^26^ (iii) reduction in processing temperatures facilitating direct incorporation of sensors which is not possible in powder bed techniques, (v) overcomes the poor mechanical properties of polymer-based parts and the sensitivity of polymeric parts to a variety of common organic solvents^17,19^.

#### Continuous Flow Copper (I) catalysed azide-alkyne cycloaddition

**Figure 5:**
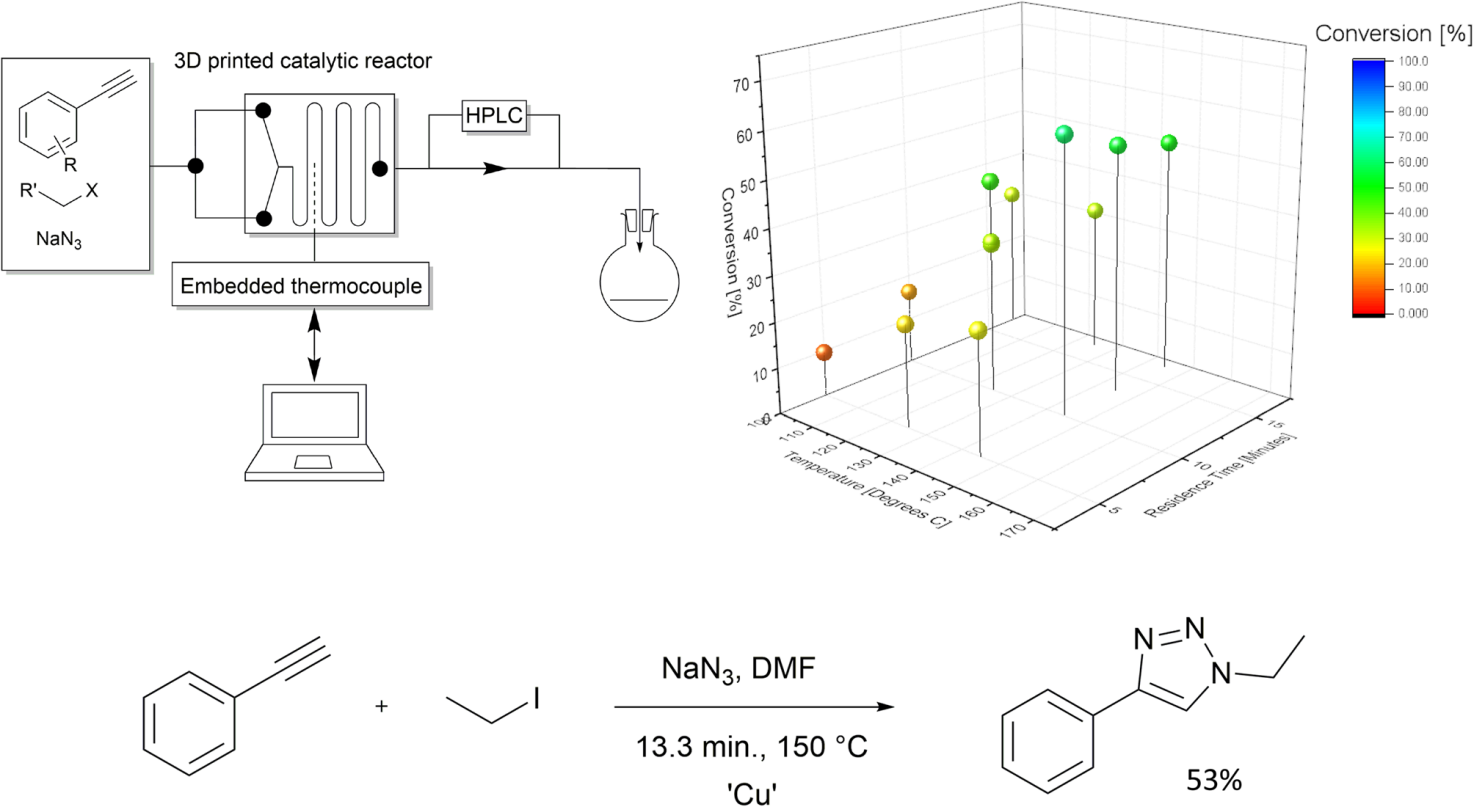
(Top left) schematic of setup used to incorporate 3DP reactor into flow system (top right) reaction conversions obtained during optimisation (bottom) scheme of Huisgen cycloaddition^57^ between Phenylacetylene and Iodoethane used in optimisation and displaying the optimised parameters.

Reactor functionality was proven through a series of copper catalysed alkyne azide cycloadditions performed under continuous flow conditions (Fig. 2). The ultrasonically printed copper reactor detailed in Fig. 4 was integrated with the commercially available flow system and employed in the library synthesis of a variety of 1,4-disubstituted 1,2,3-triazole through the temperature-controlled reaction of acetylenes and alkyl halides in the presence of sodium azide (Fig. 3). Adopting a continuous flow approach mitigated the safety concerns that may arise in a batch methodology due to this reaction generating highly reactive and dangerous azide intermediates [317], [318]. Initially, the reaction was optimised with respect to the cycloaddition of phenylacetylene and iodoethane (Scheme 1—Cycloaddition of Phenylacetylene and Iodoethane) (see Fig. 5).

Through manipulation of the reagent’s residence time within the catalytic section of the reactor, and close monitoring of the reaction temperature through the directly integrated thermocouple probe, it was possible to rapidly and accurately optimise the reaction conditions with minimal time and material consumption. It was quickly established that the highest conversion percentages were attained when using a residence time of 15 min and a reaction temperature of 150 °C. Based on the MODDE software's coefficient plots, it was possible to see that both the residence time and reaction temperatures were deemed to be significant model terms. Running an inbuilt optimisation program with these selected terms generated a set of reaction conditions intended to maximise product peak area whilst subsequently reducing starting material peak area. This optimisation yielded a conversion of 53% for the triazole product, closely matching the 54% predicted by the model.

Based upon literature suggesting that copper (I) oxide (Cu_2_O) is able to act as an effective catalytic species on zero valent copper surfaces in these reactions, the ability to pre-oxidise the surface of the reactor prior to performing the reaction in flow was investigated^70,71^. The reaction between phenylacetylene and iodoethane was then performed once again at the optimal conditions and the yields compared. This preparative procedure was seen to result in dramatic increases in starting material conversion which was calculated to be > 99%. Monitoring via HPLC however demonstrated that this conversion reduced significantly overextended reaction times until approximately 90 min, whereby the activity appears to level off and reach a 'steady state'. This observation indicates that the catalytically active source is attained from surface Cu oxide as opposed to the zero-valent Cu substrate. Cu metal is readily oxidised at room temperature to form non-self-protecting layers of CuO and Cu_2_O. This acts to negate the requirement to add a secondary Cu (II) source for co-proportionation^71^.

A major advantage of using heterogeneous catalyst sources such as solid Cu surfaces is the small amount of leached material that needs to be removed from the product, which has substantial green implications^51,54^. ICP-OES established that the average Cu concentration collected in the resulting reaction mixture was 208 ppm on average with a standard deviation of 74 ppm. This is equivalent to 200 mg of copper removed from the reactor surface per L of reaction solution. Across a 900 mm^3^ internal reactor volume, this equates to a very fine layer of copper being removed. The reactor could therefore potentially catalyse hundreds or thousands of reactions before significant degradation occurs.

**Figure 6:**
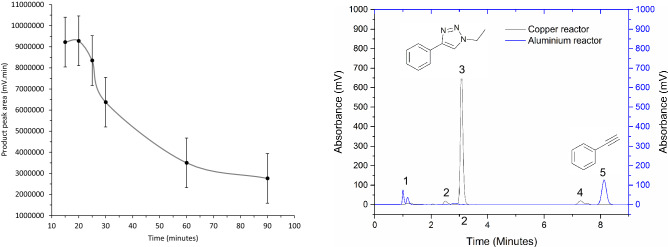
(Left) reduction in the activity of H2O2 treated Cu surfacey^72^ with time showing a steady rate of reduction until approximately 90 min (right) Comparison of Cu and Al reactors demonstrating the absence of starting material (5) in the Cu reactor and the absence of product (3) in the Al reactor.

Gas chromatography-mass spectroscopy of the resulting reaction mixture indicated that there was no presence of the undesired dialkyne by-product which can form through the oxidative coupling of alkyne species (termed the Glaser product) (Fig. 6). This coupling product forms solely in the presence of Cu (II) ions. The absence of the dialkyne indicates that that copper oxide formed within the catalytic chamber of the reaction is present as Cu_2_O only. Furthermore, to establish how this copper was liberated from the reactor surface, a solution of phenylacetylene in DMF was passed through the reactor at the prescribed conditions. The result of this was the formation of the highly coloured copper (I) acetylide. This result indicated that the terminal acetylene in the reaction mixture of the DOE study was able to coordinate with Cu (I) molecules on the reactor surface and form a preliminary reaction intermediate, thus liberating Cu and making it available for triazole formation.

**Figure 7:**
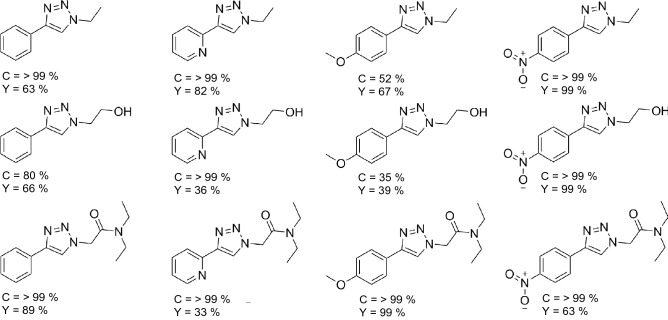
1,4-disubstituted 1,2,3-triazoles compounds synthesised using the first noted case of an ultrasonically 3D printed flow reactor. Conversions (C) were determined via HPLC analysis, whilst yields (Y) was determined via isolated yield calculations.

The reaction was repeated in an aluminium reactor of identical design and employing the optimised reaction conditions. HPLC analysis established that no reaction occurred when the catalytic copper chamber was absent from the 3DP device (Fig. 7).

Applying these conditions to the range of acetylene and alkyl halides derivatives allowed a small library synthesis to be compiled. A total of 12 compounds were prepared in a matter of hours using the optimised conditions and 3DP reactor. 

## Discussion

This copper functionalised reactor was utilised in a number of reactions all proceeding through a cuprous ion species. By chemically treating the reactor surface with an oxidising agent, a significant increase in conversion along with improvements in both yields and throughput were achieved. The reactor functionality was highlighted by the ability to perform Cu(I) mediated Huisgen cycloaddition, Castro-Stephens and Ullmann couplings through direct inclusion of a catalysing layer during the 3D printing process, and close monitoring of the reaction temperature *in-situ* via integrated thermocouples. The design versatility of UAM meant that this reactor was designed for and fitted seamlessly into a commercially available system, allowing a wide range of accurate temperatures to be utilised for reaction optimisation. As a result, it was possible to rapidly optimise a test reaction and form a small library of triazole compounds in a safe and efficient manner, despite the formation of potentially dangerous azide intermediates.

As the UAM process is a layer-by-layer process there is scope in further work to embed thermocouples at different points along the fluid path and at different points in the z-axis of the component. This would allow for an accurate understanding of the temperature evolution of the reaction through the system and allow for monitoring of heat dissipation in multiple directions around the channels. Due to the anisotropic material properties caused by the dissimilar material structure there is potentially a specific heat distribution pattern based on the different thermal conductivities of the Cu-110 and Al6061 O materials. This potential heat profile difference could also be further investigated through the creation of a device with multiple thermocouples positioned at different points in the structure. The ability to embed sensors into metallic structures is a key property of UAM that has a beneficial use in this application area.

The extensive range of materials that can be utilised for the creation of these devices via UAM has room for further exploitation. Highly chemical resistant materials such as Inconel, Stainless Steel and Titanium have all been successfully used in UAM (ref). Other materials such as Iron, Ruthenium, Nickel and Palladium have the potential to be processed via ultrasonic welding^73–75^. If they can catalyse reactions in their native metal form or be formed through additional surface modification, they could be used to form different catalytic reactors.

While the merits of flow chemistry devices are well documented, there is a continuous desire to integrate analytical tools within these systems for online process monitoring^66^^,^^67^. The ability to perform in-line reaction monitoring makes flow chemistry a powerful laboratory tool for reaction and kinetic studies. Monolithic integration of analytical sensing devices such as thermal couples and optical fibres directly into the interlaminar region of future UAM devices can provide true online analysis of the reaction mixture. Aside from fast data acquisition, online reaction monitoring can make complex analysis simpler to perform, permitting faster and more reliable process optimisation.

## Conclusion

In this study, a Cu/Al catalytic micro-reactor with embedded in-situ temperature monitoring has exploited Additive Manufacturing’s design freedoms, and the unique abilities of the Ultrasonic Additive Manufacturing (UAM) technique have been successfully designed, manufactured and utilised. Several Cu catalysed reactions in various reaction conditions were successfully performed, demonstrating these devices' functionality and versatility. The combination of greater design freedoms, unique material combinations and embedded sensors shows the emerging opportunities available to chemical processing by exploiting the quickly evolving field of AM.

## Supplementary Information

Supplementary Information.
