# Supplementary material for: Complementary catalysis and analysis within solid state additively manufactured metal micro flow reactors
Source: Sci Rep. 2022 Mar 24;12:5121. doi: 10.1038/s41598-022-09044-9 (PMC8948297; doi:10.1038/s41598-022-09044-9)
Supplement: Supplementary file 1 — Supplementary Information. [file 41598_2022_9044_MOESM1_ESM.docx]

**Supplementary Information for**

**Complementary Catalysis and Analysis within Solid State Additively Manufactured Metal Micro Flow Reactors**

T. Monaghan^a^, M. J. Harding^b^, S. D. R. Christie^c^, R. A. Harris^d^ and R. J. Friel^e*^

^a^ School of Mechanical, Electrical and Manufacturing Engineering, Loughborough University, Loughborough, United Kingdom

^b^ School of Chemical and Bioprocess Engineering, University College Dublin, Dublin, Ireland

^c^ Department of Chemistry, Loughborough University, Loughborough, United Kingdom

^d^ School of Mechanical Engineering, University of Leeds, Leeds, United Kingdom

^e^School of Information Technology, Halmstad University, Halmstad, Sweden

Author contact information: [ross.friel@hh.se](mailto:ross.friel@hh.se);

# Device Fabrication

## Aluminium 3DP Reactor

Using the same Ultrasonic Additive Manufacturing (UAM) system as that used to manufacture the Copper reactor, an Aluminium reactor of the identical design was also manufactured. This device was manufactured from a combination of Al 5052 and 6061 alloys (see Figure S 1).


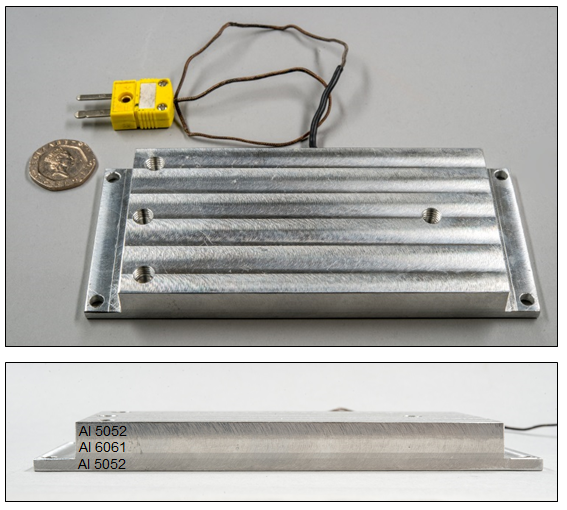

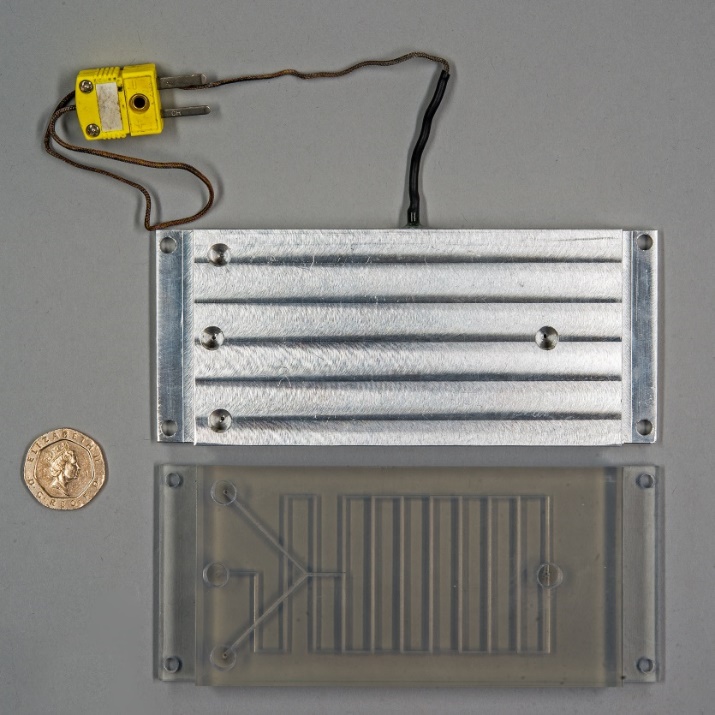


Figure S 1 - Images of the aluminium 3D-printed reactor and its SLA formed prototype

## Additional Images of Catalytic Copper Reactor

Additional images of the catalytically active Copper reactor employed in this work are shown in Figure S 2.


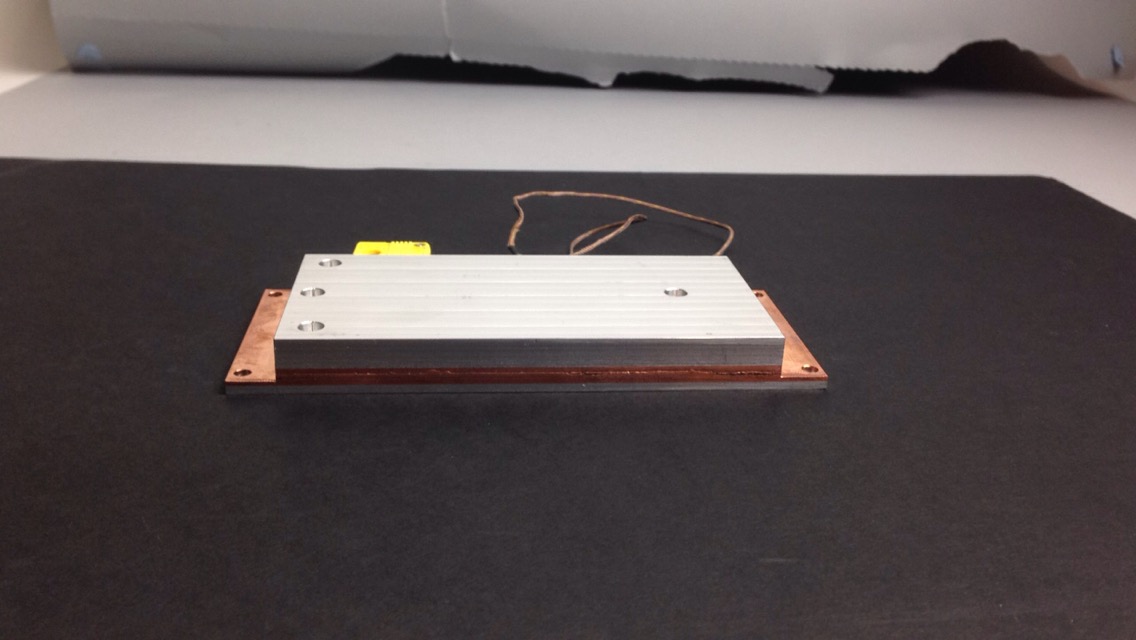

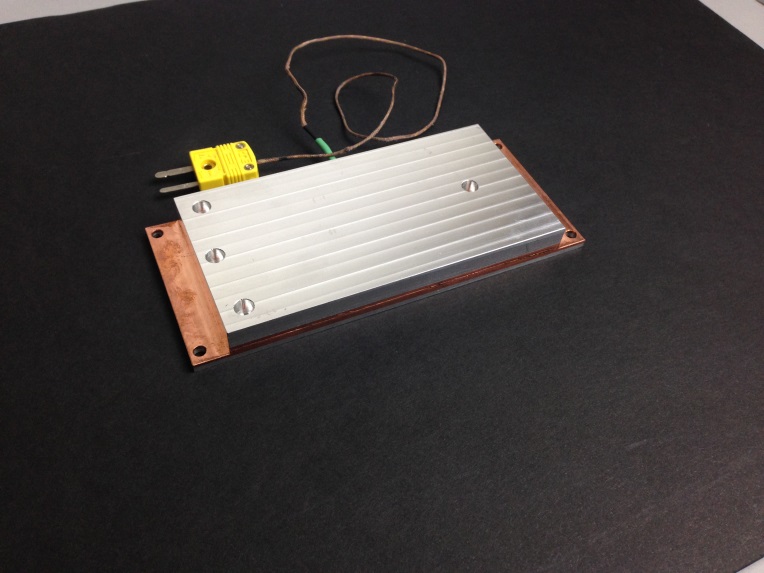

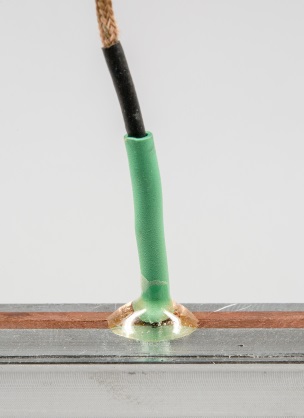


Figure S 2 - Additional images of copper flow reactor detailing the inserted thermocouple for temperature monitoring


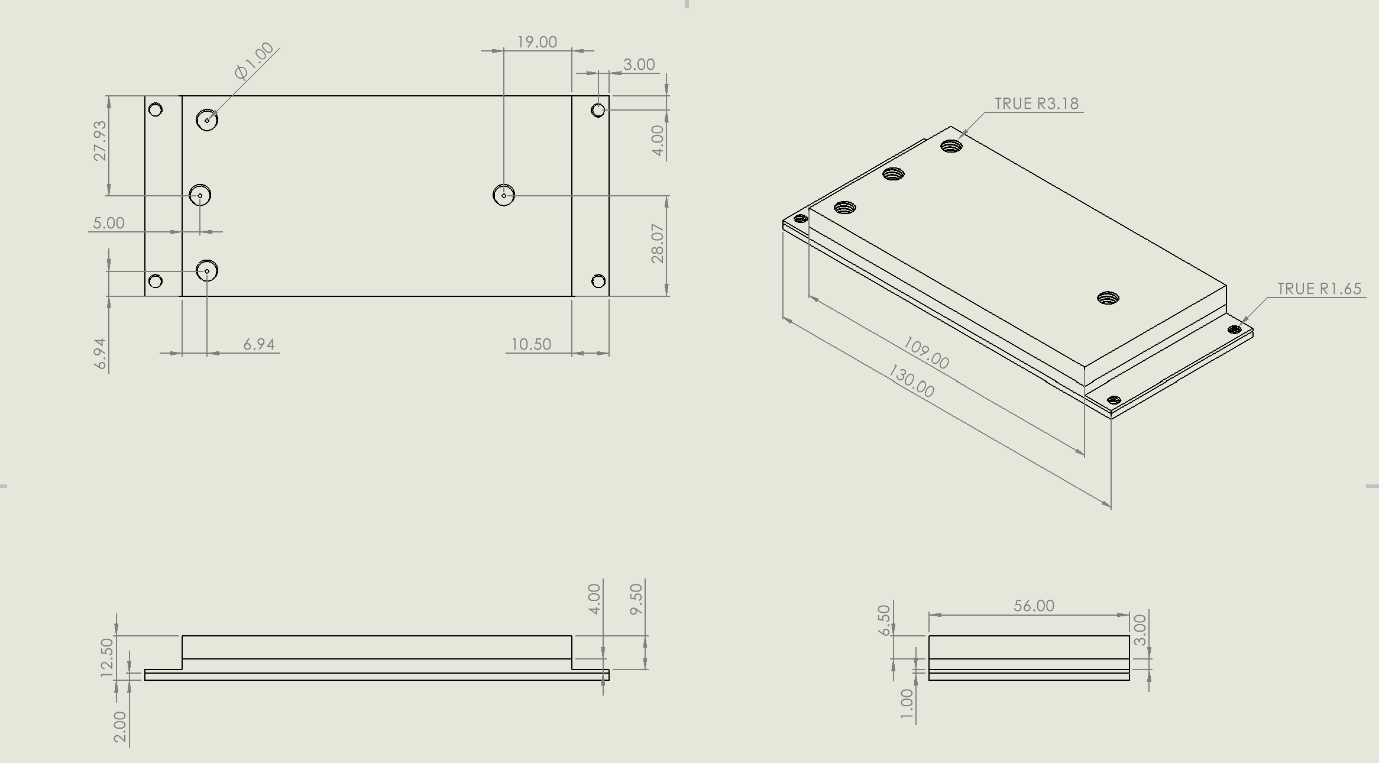


Figure S 3 - Dimensioned drawings of the copper flow reactor


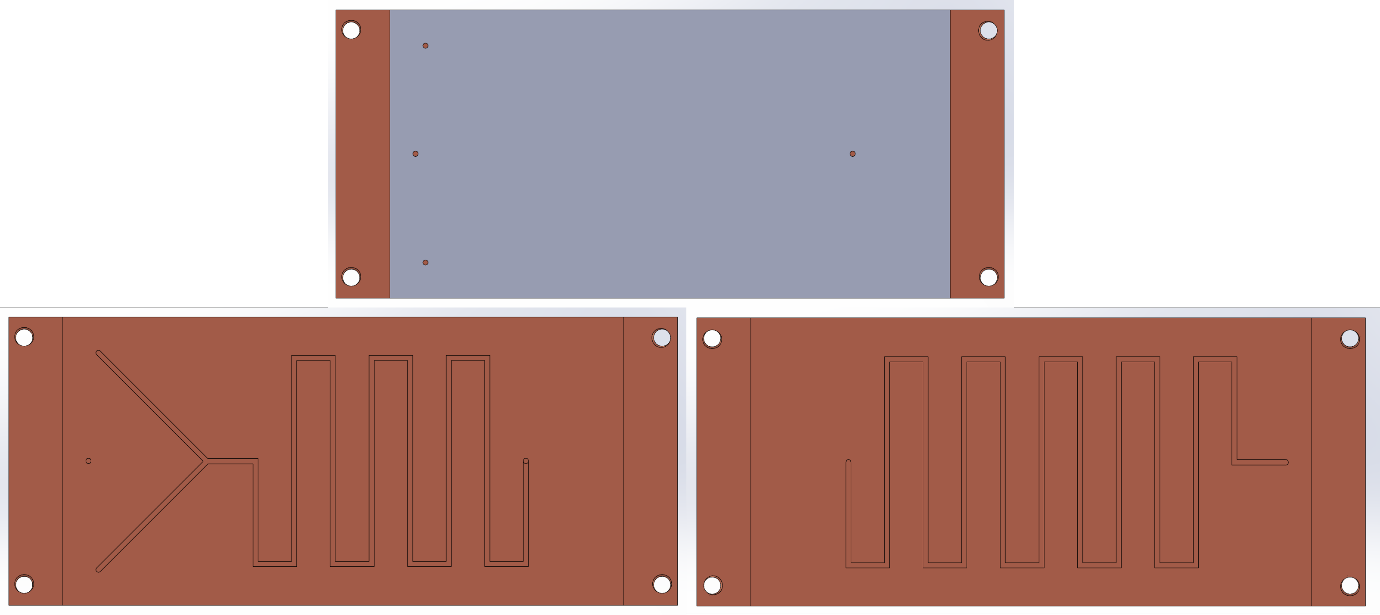


Figure S 4 - Dimensioned drawings of the copper flow reactor

# Triazole Library Design of Experiments Data

The Huisgen 1,3-dipolar cycloaddition of alkynes and azides to yield 1,2,3-triazoles is considered the premier example of a "Click Reaction". "Click Chemistry" is a term initially introduced by Professor K. B. Sharpless in 2001 to describe high yielding reactions, wide in scope, stereospecific, simple to perform, and can be conducted in easily removable or benign solvents.

## Model Reaction





Table S 1 - Conditions Screened

| Variable | Low | Medium | High |
| --- | --- | --- | --- |
| Temperature [^o^C] | 100 | 125 | 150 |
| Residence Time [min] | 5 | 10 | 15 |

Design of experiments was performed using a 3 level, full factorial design with an additional two centre point repeats for a total of 11 experimental runs.

Table S 2 - Design of Experiment Results

| Temperature  [^o^C] | Residence time  [min] | HPLC Area  % Product |
| --- | --- | --- |
| 100 | 5 | 9.69 |
| 125 | 5 | 22.37 |
| 145 | 5 | 26.26 |
| 100 | 10 | 16.25 |
| 125 | 10 | 33.24 |
| 145 | 10 | 59.57 |
| 100 | 16.7 | 30.82 |
| 125 | 16.7 | 32.03 |
| 145 | 16.7 | 50.97 |
| 125 | 10 | 32.7 |
| 125 | 10 | 46.23 |


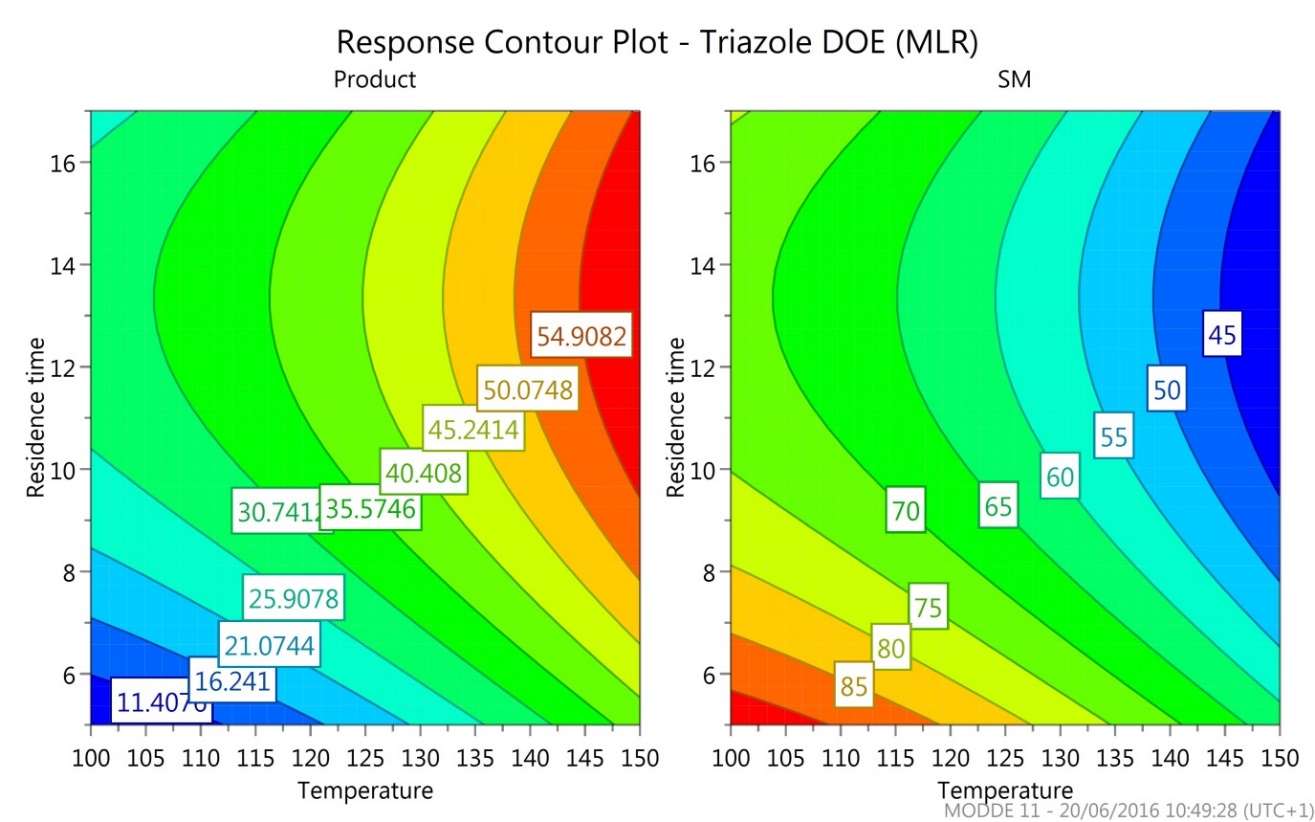


Figure S 5 - Response Contour Plot (RCP) for the optimisation design of experiments for a) product and b) starting material


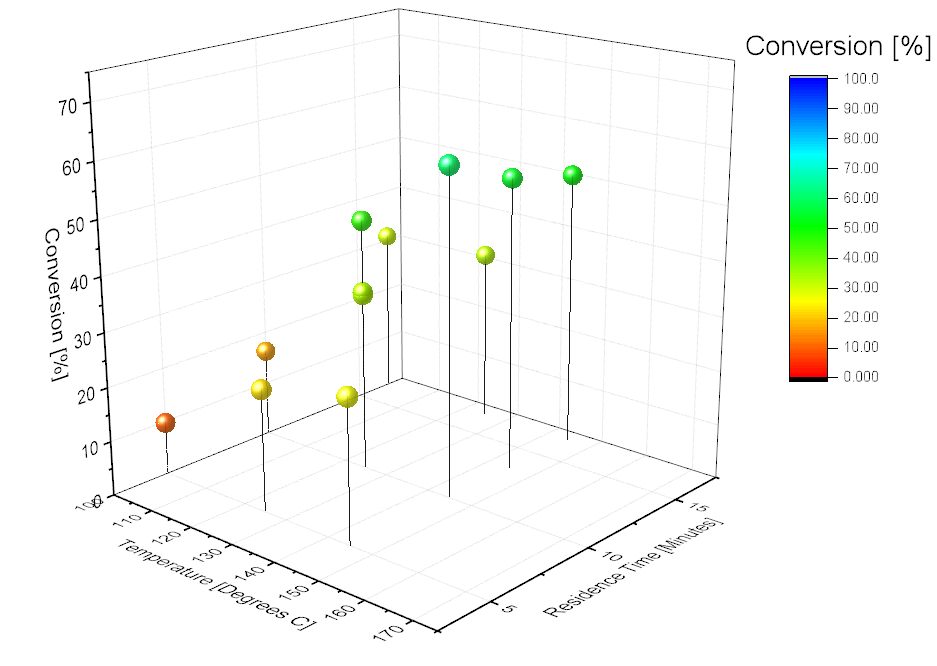


Figure S 6 - Ball and Stick diagram for individual responses in the design of experiments


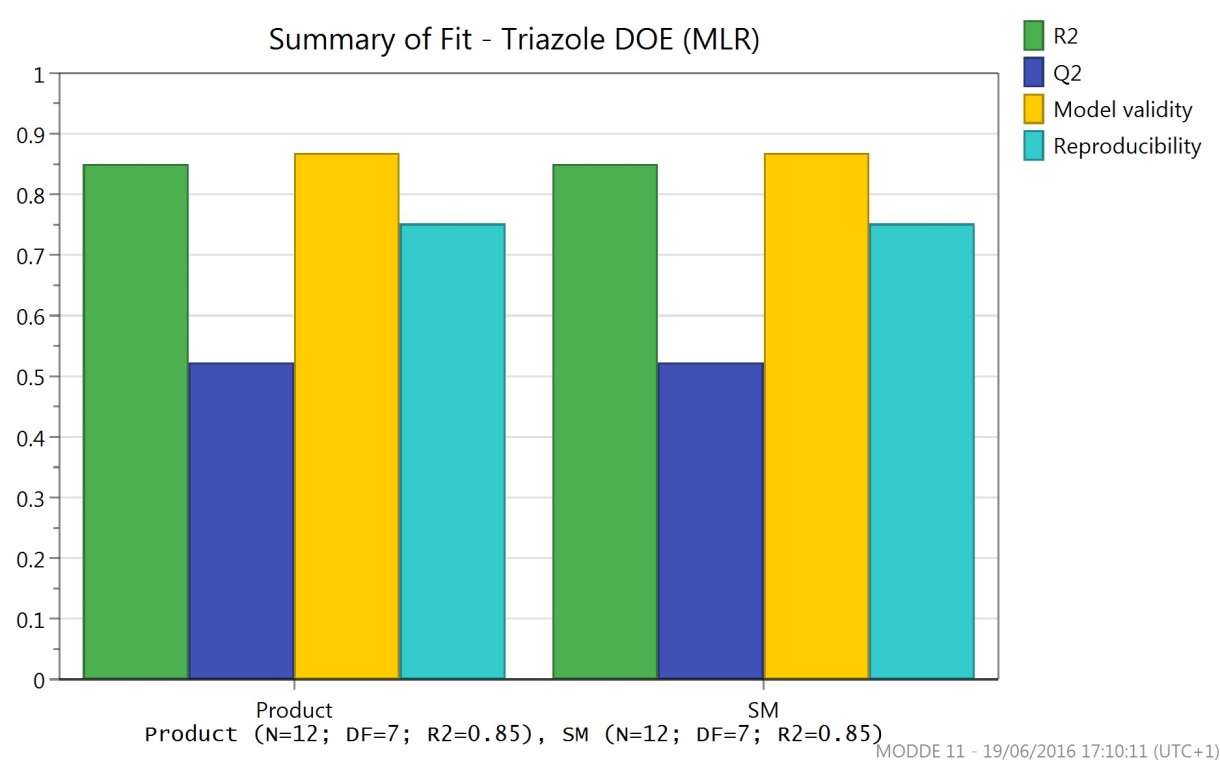


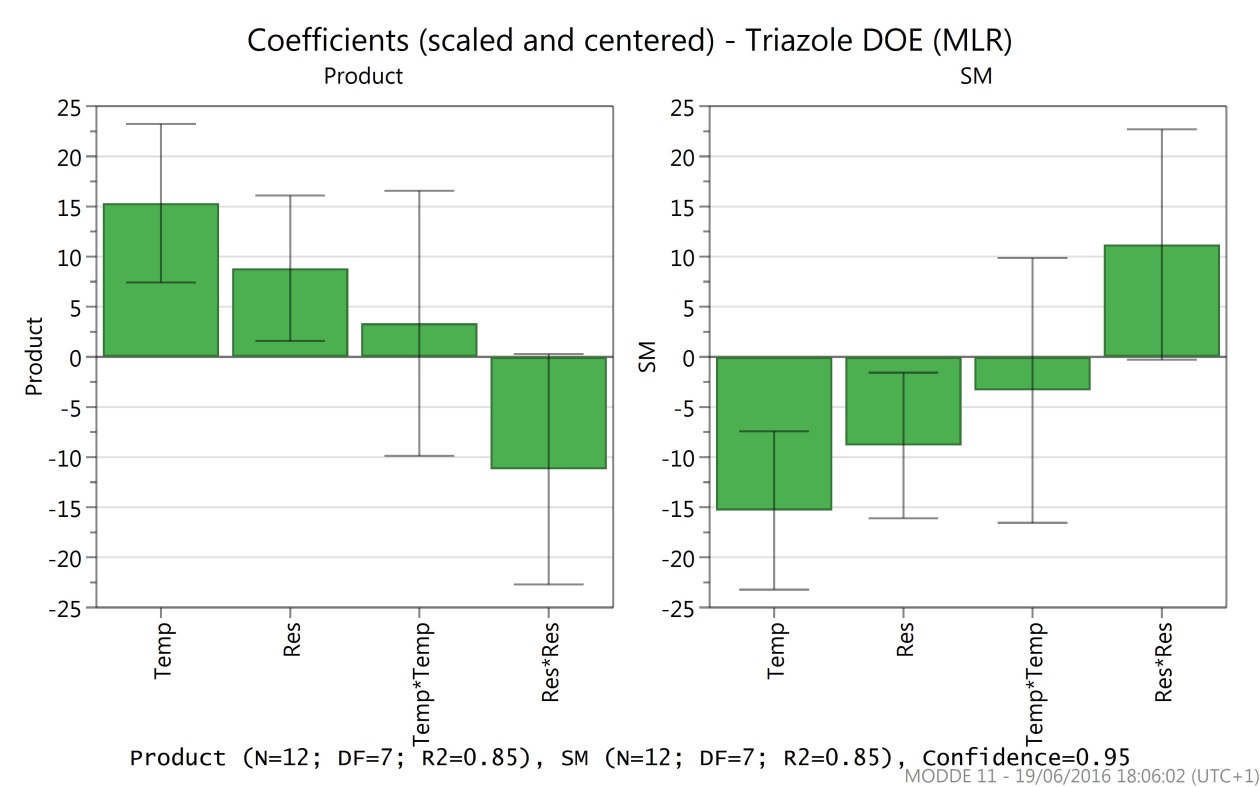
Figure S 7 - Summary of fit information for the design of experiments model

Figure S 8 - Coefficients for model generated from design of experiment results

Table S 3 - Coefficient list for product and starting material plots

| **Product** | **Coeff. SC** | **Std. Err.** | **P** | **Conf. int(±)** |
| --- | --- | --- | --- | --- |
| **Constant** | 38.8323 | 4.22064 | 9.29564e-005 | 10.3276 |
| Temperature | 15.5484 | 3.95008 | 0.00765766 | 9.66556 |
| Residence time | 8.91594 | 3.35554 | 0.0376706 | 8.21078 |
| Temp*Temp | 3.63107 | 6.33761 | 0.587498 | 15.5077 |
| Res*Res | -11.4248 | 5.45848 | 0.0812566 | 13.3565 |
|  |  |  |  |  |
|  |  |  |  |  |
| N = 11 | Q2 = | 0.361 | Cond. no. = | 3.677 |
| DF = 6 | R2 = | 0.823 | RSD = | 8.049 |
|  | R2 adj. = | 0.704 |  |  |
|  |  |  | Confidence = | 0.95 |
|  |  |  |  |  |
| **SM** | **Coeff. SC** | **Std. Err.** | **P** | **Conf. int(±)** |
| **Constant** | 61.1677 | 4.22064 | 6.76579e-006 | 10.3276 |
| Temperature | -15.5484 | 3.95007 | 0.00765764 | 9.66556 |
| Residence time | -8.91595 | 3.35554 | 0.0376706 | 8.21077 |
| Temp*Temp | -3.63107 | 6.33761 | 0.587498 | 15.5077 |
| Res*Res | 11.4248 | 5.45848 | 0.081257 | 13.3565 |
|  |  |  |  |  |
|  |  |  |  |  |
| N = 11 | Q2 = | 0.361 | Cond. no. = | 3.677 |
| DF = 6 | R2 = | 0.823 | RSD = | 8.049 |
|  | R2 adj. = | 0.704 |  |  |
|  |  |  | Confidence = | 0.95 |

All summary terms are of suitable magnitude to suggest that the DOE model generated is good and able to be used with a high degree of reliability. Data suggest that higher temperatures and moderate residence times are favourable for achieving high degrees of product conversion. P values indicate that the model in linear, as the squared terms Temp*Temp and Res*Res are <0.05 and therefore these 2^nd^ order effects are insignificant. Using inbuilt optimisation software in the MODDE Pro software generated the following conditions for maximising product conversion and minimising starting materials;

Table S 4 - Optimised Conditions based on Design of Experiments Model

| Temperature  [^o^C] | Residence Time  [minutes] | Predicted Product Conversion [%] | Obtained Product Conversion [%] |
| --- | --- | --- | --- |
| 150 | 13.3 | 55.3 | 53.9 |

* The close match between predicted and observed values signify the high reliability of the generated model

## Hydrogen Peroxide Treatment

In order to fully maximise yields, an additional pre-run was performed in which hydrogen peroxide (36%) was flowed through the reaction chamber. The optimised reaction conditions were then employed to establish if this peroxidation could increase conversions. Whilst all results yielded significant increases in product conversion, a slight drop off was observed after 2.5 minutes, and this was therefore used as the minimum exposure time (Table S 5).

Table S 5 - Results of Peroxide surface treatment

| Temperature  [^o^C] | Residence Time  [minutes] | Hydrogen Peroxide Pre-run Residence Time [minutes] | Product Conversion [%] |
| --- | --- | --- | --- |
| 150 | 13.3 | 0.5 | 91 |
| 150 | 13.3 | 1.0 | 93 |
| 150 | 13.3 | 2.5 | > 95 |
| 150 | 13.3 | 5.0 | > 95 |
| 150 | 13.3 | 10 | > 95 |
| 150 | 13.3 | 15 | > 95 |
| 150 | 13.3 | 30 | > 95 |

# Library Synthesis Procedure

## Terminal Acetylenes Used





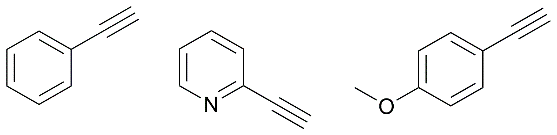

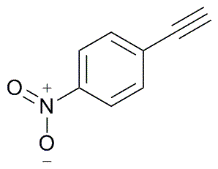


## Alkyl Halides Used

4-Ethynyltoluene (133.3 mL, 0.25 M in DMF, 0.033 mmol, 1.0

equiv.), ethyl iodide (133.3 mL, 0.5 M in DMF, 0.066 mmol,

2.0 equiv.), and NaN

3

(133.3 mL, 0.5 M in DMF/H

2

O 4:1 v/v,

0.066 mmol, 2.0 equiv.) were aspirated from their respective

source *via*ls, mixed through a PFA mixing tube (0.2 mm

i. d.), loaded into an injection loop and injected into the

flow reactor set at 150

8C at a rate of 400 mLmin

1

(5 min

residence time). Reaction segments were collected in a 96-

well plate containing QuadraPure TU copper-scavenging

resin. Segments were filtered, concentrated, and analysed

using

1

H NMR.

4-Ethynyltoluene (133.3 mL, 0.25 M in DMF, 0.033 mmol, 1.0

equiv.), ethyl iodide (133.3 mL, 0.5 M in DMF, 0.066 mmol,

2.0 equiv.), and NaN

3

(133.3 mL, 0.5 M in DMF/H

2

O 4:1 v/v,

0.066 mmol, 2.0 equiv.) were aspirated from their respective

source *via*ls, mixed through a PFA mixing tube (0.2 mm

i. d.), loaded into an injection loop and injected into the

flow reactor set at 150

8C at a rate of 400 mLmin

1

(5 min

residence time). Reaction segments were collected in a 96-

well plate containing QuadraPure TU copper-scavenging

resin. Segments were filtered, concentrated, and analysed

using

1

H NMR.

Separate solutions of sodium azide (0.25 M, 4:1 DMF:H_2_O) iodoethane (0.25 M, DMF) and phenyl acetylene (0.125 M, DMF) were prepared. 3 mL aliquots of each solution were mixed and pumped through the reactor at 75 µL.min^-1^ and 145 °C. The total volume was collected into a *via*l and diluted with 10 mL ethyl acetate. The sample solution was washed with 3 x 10 mL water. The aqueous layers were combined and extracted with 10 mL ethyl acetate, the organic layers were then combined, washed with 3 x 10 mL brine, dried with MgSO_4_ and filtered before the solvent was removed *in vaccuo.* Samples were purified by column chromatography on silica gel using ethyl acetate prior to analysis *via* NMR, high-resolution mass spectroscopy and HPLC.

# Triazole Library Synthesis Characterisation Data

## 1-Ethyl-4-phenyl-1*H*-1,2,3-triazole

^1^H NMR (CDCl_3_ 400 MHz) δ 1.59 (t, 3H, *J* = 7 Hz), 4.45 (q, 2H, *J* = 7 Hz), 7.33-7.35 (m, 1H), 7.40-7.44 (m, 2H), 7.77 (s, 1H), 7.82-7.84 (m, 2H); ^13^C NMR (CDCl_3_ 100 MHz) δ 15.8, 45.4, 119.0, 125.9, 128.3, 129.0, 130.9, 148.1; HRMS: *m/z* calculated for C_10_H_11_N_3_Na [M+Na]^+^ 196.0851; found: 196.0844.

## 2-(4-Phenyl-1*H*-1,2,3-triazol-1-yl)ethan-1-ol

^1^H NMR (CDCl_3_ 400 MHz) δ 4.14 (t, 2H, *J* = 5 Hz), 4.54 (t, 2H, *J* = 5 Hz), 7.33 (t, 1H, *J* = 7 Hz), 7.41 (t, 2H, *J* = 7 Hz), 7.76 (d, 2H, *J* = 7 Hz), 7.85 (s, 1H); ^13^C NMR (CDCl_3_ 100 MHz) δ 52.9, 61.5, 121.1, 125.8, 128.4, 129.0, 130.5, 147.8; HRMS: *m/z* calculated for C_10_H_11_N_3_ONa [M+Na]^+^ 212.0800; found *m/z* 212.0792.

## *N,N*-diethyl-2-(4-phenyl-1*H*-1,2,3-triazol-1-yl)acetamide

^1^H NMR (CDCl_3_ 400 MHz) δ 1.15 (t, 3H, *J* = 7 Hz), 1.26 (t, 3H, *J* = 7 Hz), 3.40-3.47 (m, 4H), 5.24 (s, 2H), 7.30-7.34 (m, 1H), 7.40-7.44 (m, 2H), 7.82-7.86 (m, 2H), 8.04 (s, 1H); ^13^C NMR (CDCl_3_ 100 MHz) δ 13.0, 14.6, 41.2, 42.2, 51.1, 121.6, 125.9, 128.3, 129.0, 130.7, 148.2, 164.2; HRMS: *m/z* calculated for C_14_H_18_N_4_ONa [M+Na]^+^ 281.1378; found *m/z* 281.1369.

## 1-Ethyl-4-(4-methoxyphenyl)-1*H*-1,2,3-triazole

^1^H NMR (CDCl_3_ 400 MHz) δ 1.59 (t, 3H, *J* = 7 Hz), 3.84 (s, 3H), 4.44 (q, 2H, *J* = 7 Hz), 6.96 (d, 2H, *J* = 9 Hz), 7.69 (s, 1H), 7.75 (d, 2H, *J* = 9 Hz); ^13^C NMR (CDCl_3_ 100 MHz) δ 15.8, 45.5, 55.5, 114.4, 118.3, 127.2, 147.9, 159.7; HRMS: *m/z* calculated for C_11_H_13_N_3_ONa [M+Na]^+^ 226.0956; found *m/z* 226.0949.

## 2-(4-(Methoxyphenol)-1H-1,2,3-triazol-1-yl)ethan-1-ol

^1^H NMR (CDCl_3_ 400 MHz) δ 3.84 (s, 3H), 4.13 (t, 2H, *J* = 5 Hz), 4.45 (t, 2H, *J* = 5 Hz), 6.89 (d, 2H, *J* = 9 Hz), 7.67 (d, 2H, *J* = 9 Hz), 7.74 (s, 1H); ^13^C NMR (CDCl_3_ 100 MHz) δ 52.9, 55.5, 61.5, 114.4, 120.3, 123.2, 127.1, 147.6, 159.8; HRMS: *m/z* calculated for C_11_H_13_N_3_O_2_Na [M+Na]^+^ 242.0905; found *m/z* 242.0897.

## *N,N*-diethyl-2-(4-(-methoxyphenyl)-1*H*-1,2,3-triazol-1-yl)acetamide

^1^H NMR (CDCl_3_ 400 MHz) δ 1.12 (t, 3H, *J* = 7 Hz), 1.23 (t, 3H, *J* = 7 Hz), 3.40 (q, 4H, *J* = 7 Hz), 3.82 (s, 2H), 5.20 (s, 2H), 6.93 (d, 2H, *J* = 9 Hz), 7.75 (d, 2H, *J* = 9 Hz), 7.92 (s, 1H); ^13^C NMR (CDCl_3_ 100 MHz) δ 13.0, 14.6, 41.2, 42.2, 51.2, 55.5, 114.4, 120.8, 123.4, 127.3, 148.0, 159.8, 164.2; HRMS: *m/z* calculated for C_15_H_20_N_4_O_3_Na [M+Na]^+^ 311.1484; found *m/z* 311.1474.

## 2-(1-Ethyl-1*H*-1,2,3-triazol-4-yl)pyridine

^1^H NMR (CDCl_3_ 400 MHz) δ 1.60 (t, 3H, *J* = 7 Hz), 4.48 (q, 2H, *J* = 7 Hz), 7.20-7.24 (m, 1H), 7.75-7.80 (m, 1H), 8.15 (s, 1H) 8.15-8.20 (m, 1H), 8.55-8.59 (m, 1H); ^13^C NMR (CDCl_3_ 100 MHz) δ 15.6, 45.7, 12.4, 121.5, 123.0, 137.2, 148.6, 149.5, 150.5; HRMS: *m/z* calculated for C_9_H_10_N_4_Na [M+Na]^+^ 197.0803; found *m/z* 197.0797.

## 2-(4-(Pyridine-2-yl)-1*H*-1,2,3-triazol-1-yl)ethan-1-ol

^1^H NMR (CDCl_3_ 400 MHz) δ 4.13 (t, 2H, *J* = 5 Hz), 4.56 (t, 2H, *J* = 5 Hz), 7.21 (t, 1H, *J* = 5 Hz), 7.75 (t, 1H, *J* = 8 Hz), 8.07 (d, 1H, *J* = 8 Hz), 8.30 (s, 1H), 8.46 (d, 1H, *J* = 5 Hz); ^13^C NMR (CDCl_3_ 100 MHz) δ 53.2, 61.3, 120.5, 123.1, 123.7, 137.4, 147.8, 149.2, 150.1; HRMS: *m/z* calculated for C_9_H_10_N_4_ONa [M+Na]^+^ 213.0752; found *m/z* 213.0745.

## *N,N*-diethyl-2-(4-(pyridine-2-yl)-1*H*-1,2,3-triazol-1-yl)acetamide

^1^H NMR (CDCl_3_ 400 MHz) δ 1.14 (t, 3H, *J* = 7 Hz), 1.26 (t, 3H, *J* = 7 Hz), 3.42 (m, 4H), 5.25 (s, 2H), 7.19-7.23 (m, 1H), 7.74-7.78 (m, 1H), 8.14 (d, 1H, *J* = 8 Hz), 8.35 (s, 1H), 8.57 (d, 1H, *J* = 5 Hz); ^13^C NMR (CDCl_3_ 100 MHz) δ 13.0, 14.6, 41.2, 42.1, 51.2, 120.5, 123.0, 124.1, 137.0, 148.8, 149.6, 150.4, 163.9; HRMS: *m/z* calculated for C_13_H_17_N_5_ONa [M+Na]^+^ 282.1331; found *m/z* 282.1320.

## 1-Ethyl-4-(4-nitrophenyl)-1*H*-1,2,3-triazole

^1^H NMR ((CD_3_)_2_SO 400 MHz) δ 1.52 (t, 3H, *J* = 7 Hz), 4.49 (q, 2H, *J* = 7 Hz), 8.14 (d, 2H, *J* = 8 Hz), 8.35 (d, 2H, *J* = 8 Hz), 8.90 (s, 1H); ^13^C NMR (CDCl_3_ 100 MHz) δ 15.7, 45.8, 120.6, 124.5, 126.3, 137.2, 145.9, 147.5; HRMS: *m/z* calculated for C_10_H_11_N_4_O_2_ [M+H]^+^ 219.0877; found *m/z* 219.0876.

## 2-(4-(4-Nitrophenyl)-1*H*-1,2,3-triazol-1-yl)ethan-1-ol

^1^H NMR ((CD_3_)_2_SO 400 MHz) δ 3.86 (t, 2H, *J* = 6 Hz), 4.50 (t, 2H, *J* = 6 Hz), 5.14 (s, br, 1H), 8.16 (d, 1H, *J* = 9 Hz), 8.34 (d, 1H, *J* = 9 Hz); ^13^C NMR ((CD_3_)_2_SO 100 MHz) δ 52.7, 59.7, 124.0, 124.4, 125.9, 137.4, 144.2, 146.5; HRMS: *m/z* calculated for C_10_H_11_N_4_O_3_ [M+H]^+^ 235.0826; found *m/z* 235.0824.

## *N,N*-diethyl-2-(4-(4-nitrophenyl)-1*H*-1,2,3-triazol-1-yl)acetamide

^1^H NMR (CDCl_3_ 400 MHz) δ 1.17 (t, 3H, *J* = 7 Hz), 1.31 (t, 3H, *J* = 7 Hz), 3.41-3.50 (m, 4H), 8.02 (d, 2H, *J* = 9 Hz), 8.21 (s, 1HH), 8.28 (d, 2H, *J* = 9 Hz); ^13^C NMR (CDCl_3_ 100 MHz) δ 12.8, 14.4, 41.2, 42.0, 50.8, 123.1, 124.3, 126.2, 136.9, 145.8, 147.3, 163.6; HRMS: *m/z* calculated for C_14_H_17_N_5_O_3_Na [M+Na]^+^ 326.1229; found *m/z* 326.1219.

# H NMR Spectra for Previously Unreported Compounds

## N,N-diethyl-2-(4-(pyridine-2-yl)-1*H*-1,2,3-triazol-1-yl)acetamide


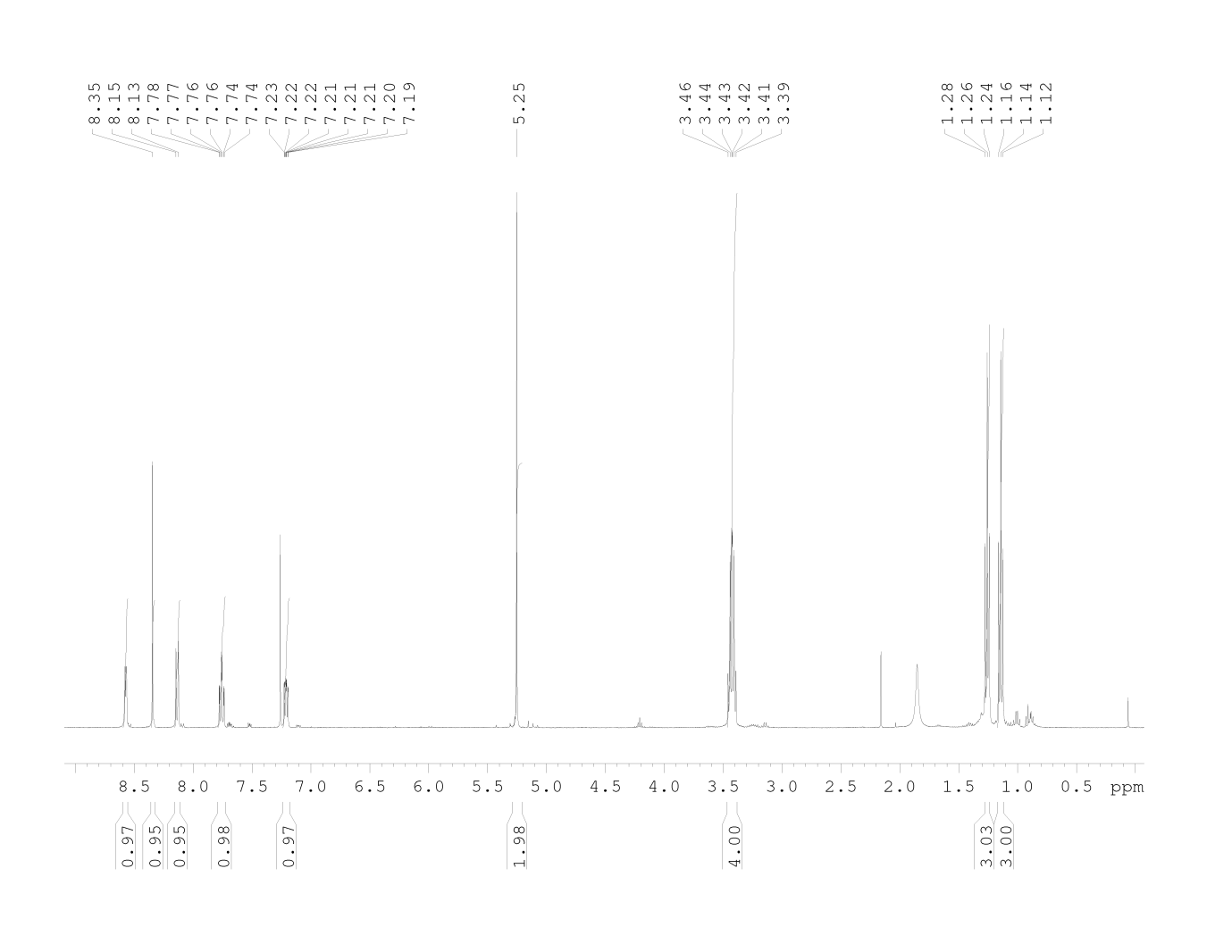


## 1-Ethyl-4-(4-methoxyphenyl)-1*H*-1,2,3-triazole


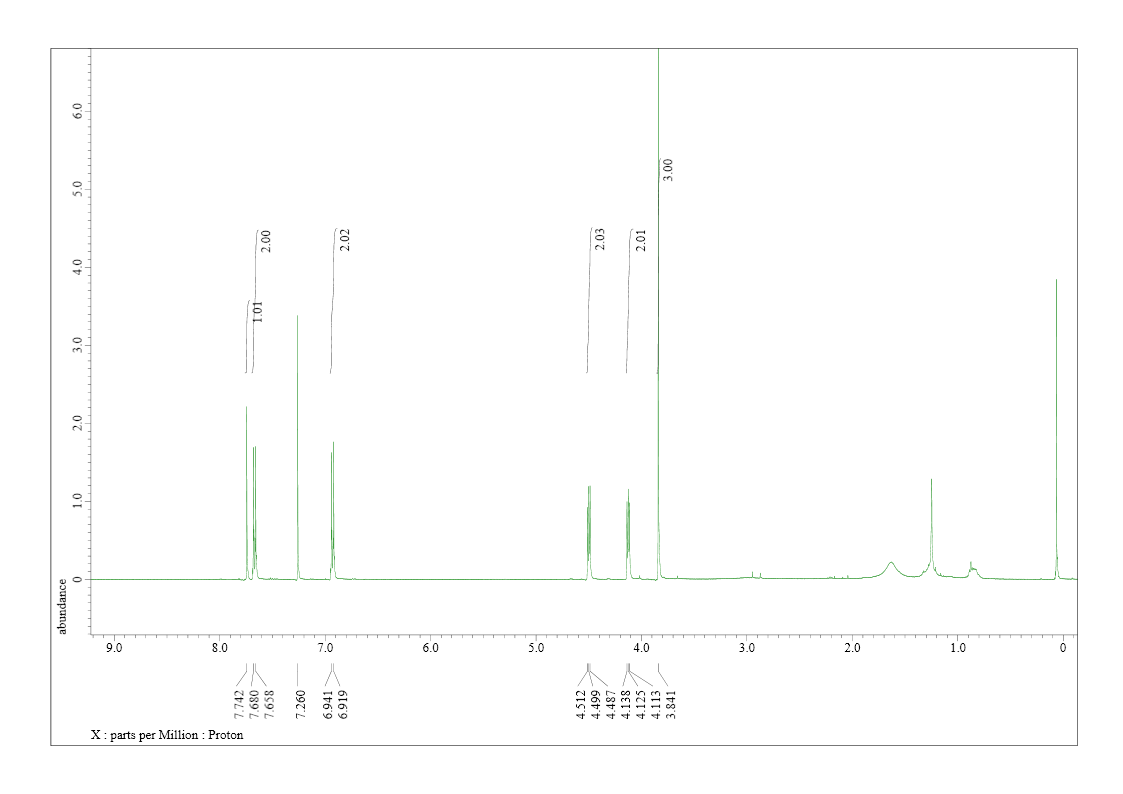


## N,N-diethyl-2-(4-(-methoxyphenyl)-1*H*-1,2,3-triazol-1-yl)acetamide


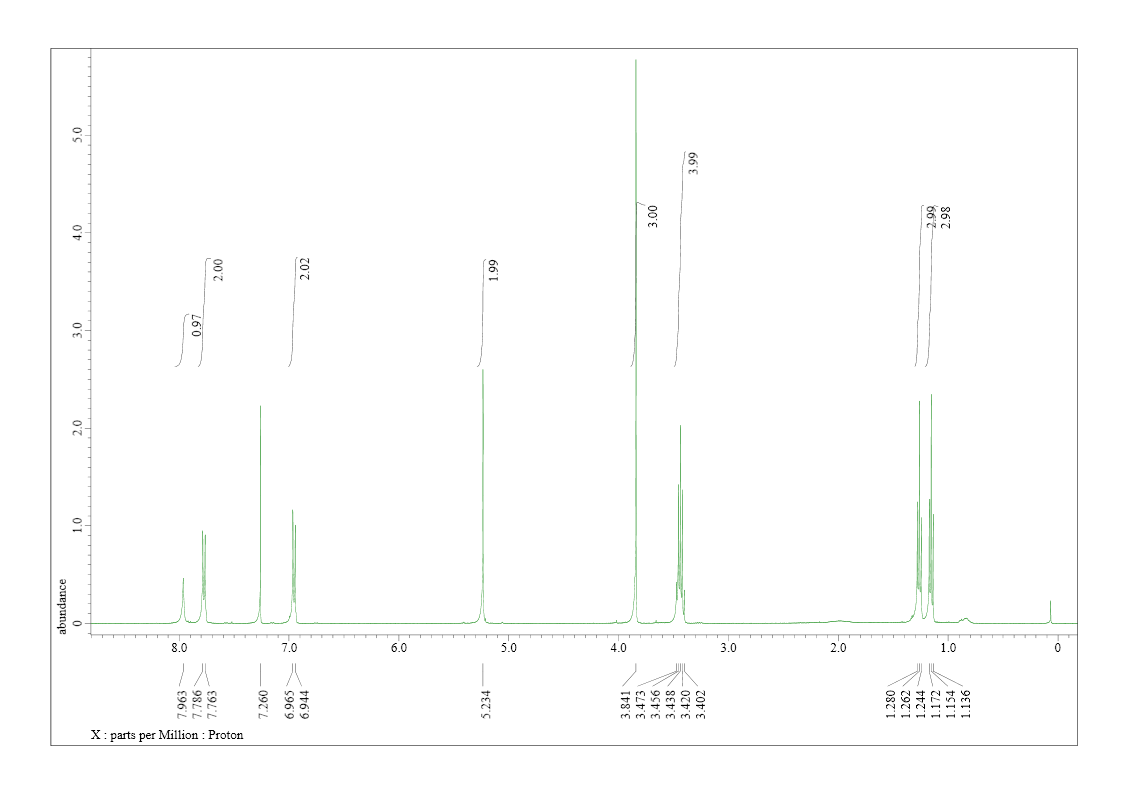


## 2-(4-(4-nitrophenyl)-1*H*-1,2,3-triazol-1-yl)ethan-1-ol


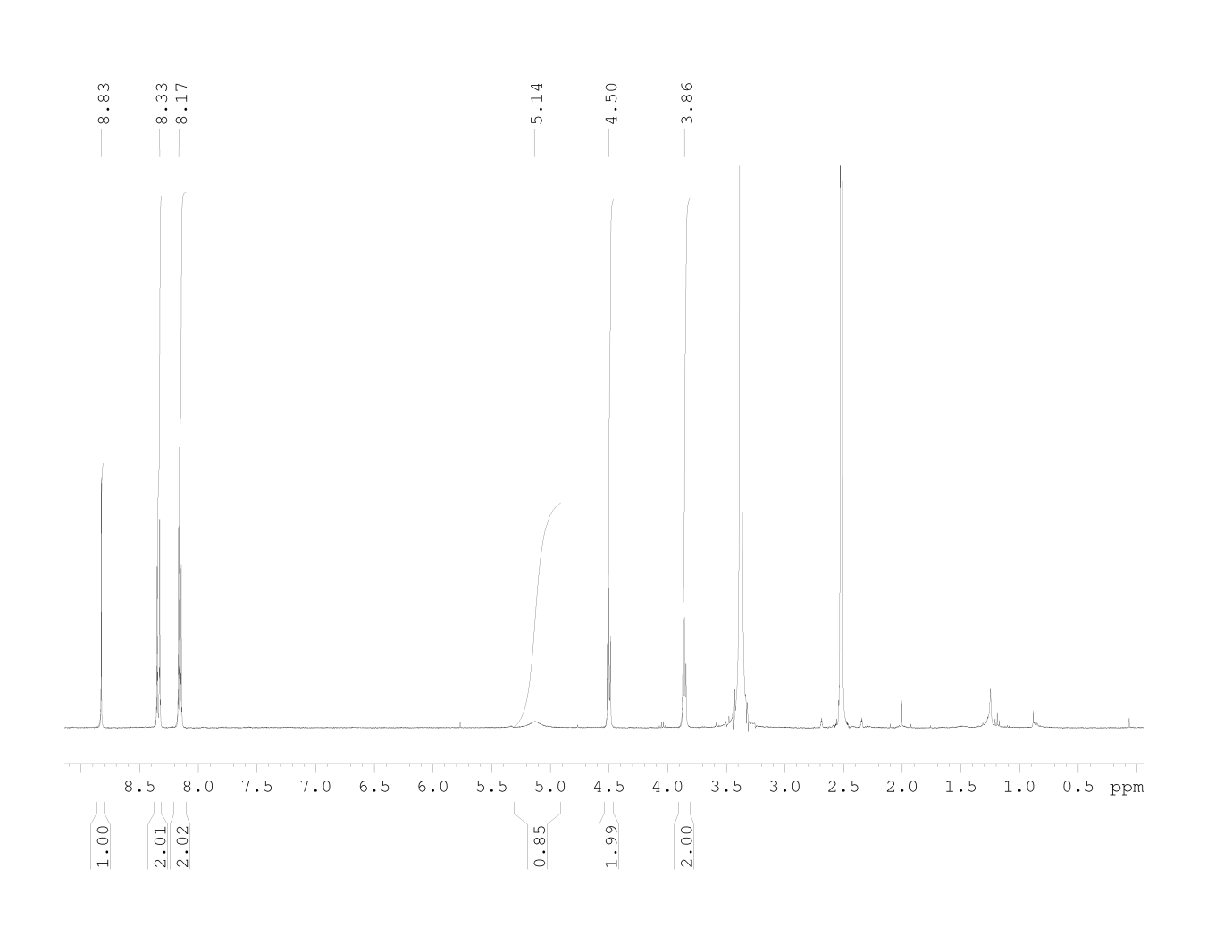


## *N,N*-diethyl-2-(4-(4-nitrophenyl)-1*H*-1,2,3-triazol-1-yl)acetamide


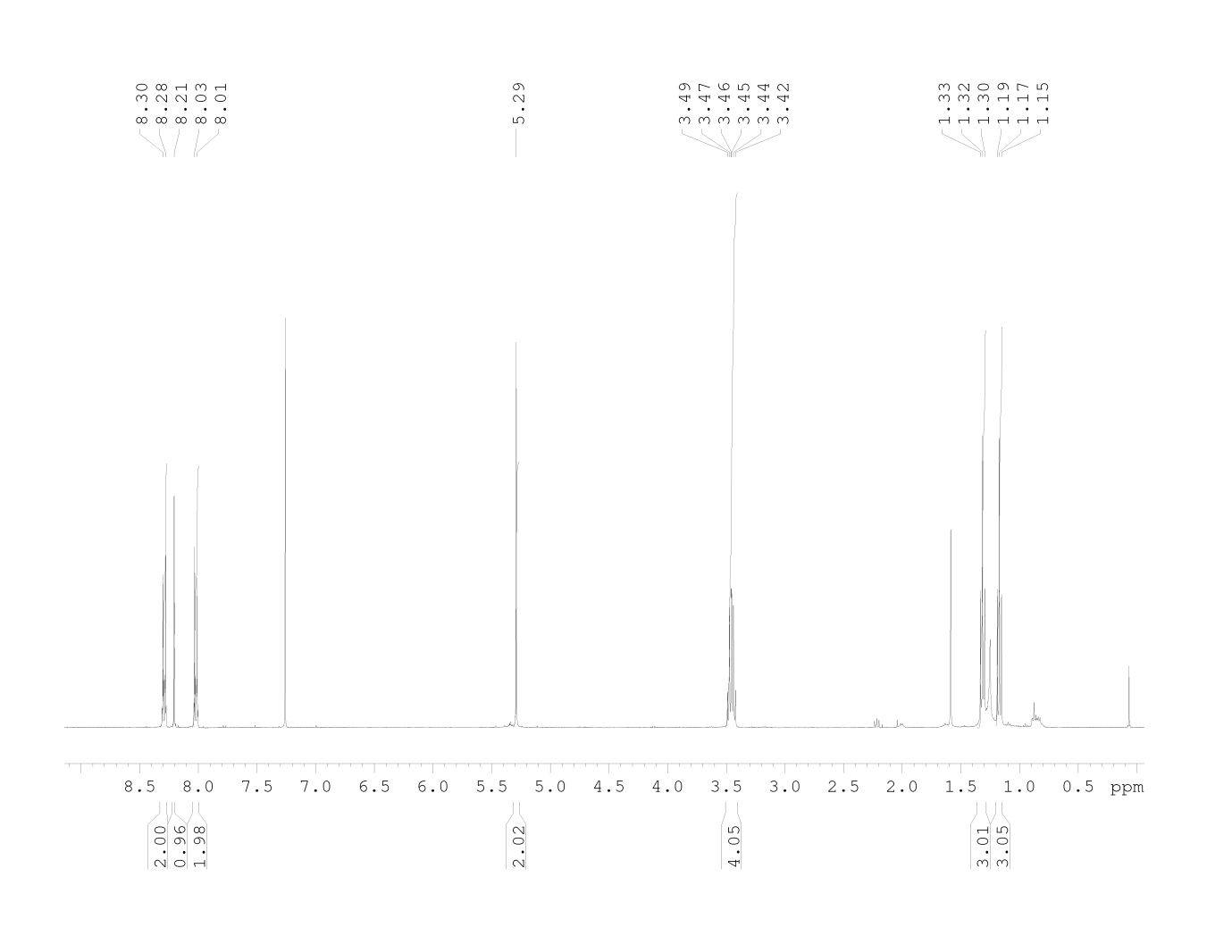


# Figure Captions

[Figure S 1 - Images of the aluminium 3D-printed reactor and its SLA formed prototype 1](#_Toc93654222)

[Figure S 2 - Additional images of copper flow reactor detailing the inserted thermocouple for temperature monitoring 2](#_Toc93654223)

[Figure S 3 - Dimensioned drawings of the copper flow reactor 3](#_Toc93654224)

[Figure S 4 - Dimensioned drawings of the copper flow reactor 3](#_Toc93654225)

[Figure S 5 - Response Contour Plot (RCP) for the optimisation design of experiments for a) product and b) starting material 6](#_Toc93654226)

[Figure S 6 - Ball and Stick diagram for individual responses in the design of experiments 6](#_Toc93654227)

[Figure S 7 - Summary of fit information for the design of experiments model 7](#_Toc93654228)

[Figure S 8 - Coefficients for model generated from design of experiment results 7](#_Toc93654229)

# Table Captions

[Table S 1 - Conditions Screened 4](#_Toc93654287)

[Table S 2 - Design of Experiment Results 5](#_Toc93654288)

[Table S 3 - Coefficient list for product and starting material plots 8](#_Toc93654289)

[Table S 4 - Optimised Conditions based on Design of Experiments Model 9](#_Toc93654290)

[Table S 5 - Results of Peroxide surface treatment 9](#_Toc93654291)
